# Nanoparticle‐Mediated CXCL12–CXCR4 Inhibition Reprograms Macrophages and Suppresses Gastric Carcinoma

**DOI:** 10.1002/advs.202500225

**Published:** 2025-06-19

**Authors:** Qianqian Cao, Xiaolei Cheng, Rongbin Lv, Dianshui Sun, Jihua Wang, Runjia Fu, Rumei Gong, Yueying Xiao, Qin Liu, Xiaomei Li

**Affiliations:** ^1^ Cancer Center, The Second Hospital Cheeloo College of Medicine Shandong University Jinan 250033 P. R. China; ^2^ Department of Oncology Qingdao Central Hospital University of Health and Rehabilitation Sciences (Qingdao Central Hospital) Qingdao 266042 P. R. China; ^3^ Postdoctoral workstation Liaocheng People's Hospital No. 67, Dongchang West Road Liaocheng 252000 P. R. China; ^4^ Department of Spine Surgery The Second Hospital Cheeloo College of Medicine Shandong University Jinan 250033 P. R. China; ^5^ Tumor Research and Therapy Center Shandong Provincial Hospital Affiliated to Shandong First Medical University Jinan 250021 P. R. China

**Keywords:** CXCL12–CXCR4 signaling pathway, gastric carcinoma, immunotherapy, M2pep‐Cs NPs/plerixafor nanoparticles, macrophage reprogramming

## Abstract

Gastric carcinoma (GC) remains a major global health challenge, requiring novel therapeutic approaches. This study investigates the efficacy of self‐assembled M2pep‐Cs NPs/Plerixafor nanoparticles in suppressing GC by targeting the CXCL12–CXCR4 signaling pathway and reprogramming tumor‐associated macrophages (TAMs) to enhance anti‐tumor immunity. The nanoparticles’ physicochemical properties and biocompatibility are assessed using transmission electron microscopy, dynamic light scattering, and biological assays. A GC mouse model is established, followed by histological and immunohistochemical analyses to evaluate tumor apoptosis and proliferation. Multi‐omics approaches, including transcriptomics, proteomics, and metabolomics, identify key genes and pathways affected by treatment. Flow cytometry and ELISA quantify immune activation markers; while, cell migration and invasion assays evaluate tumor suppression effects. The results demonstrate that M2pep‐Cs NPs/Plerixafor effectively modulates the tumor microenvironment, suppressing GC progression by reprogramming TAMs through CXCL12–CXCR4 inhibition, enhancing immune recognition and T cell responses. This study provides mechanistic insights and highlights the potential of nanoparticle‐based immunotherapy for GC, offering a promising avenue for clinical translation.

## Graphical abstract

Molecular mechanism study of self‐assembled M2pep‐Cs NPs/Plerixafor nanoparticles in targeting MΦ reprogramming by regulating the CXCL12–CXCR4 signaling pathway to enhance immune recognition and clearance of GC (Created by BioRender).

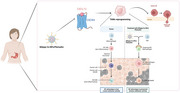

## 1 Introduction

Gastric carcinoma (GC) is the fifth most commonly diagnosed malignancy globally and ranks as the third leading cause of cancer‐related mortality.^[^ ^1^, ^2^, ^3^ ^]^ According to the World Health Organization, ≈1 million new cases of GC are diagnosed each year, resulting in up to 7 30 000 deaths annually.^[^ ^4^, ^5^, ^6^ ^]^ A major challenge in GC management lies in its frequent late‐stage diagnosis by which time the disease has often progressed to locally advanced or metastatic stages.^[^ ^7^, ^8^ ^]^ Current standard therapies—including surgical resection, chemotherapy, and radiotherapy—offer limited efficacy, especially in advanced GC, where the 5‐year survival rate remains dismally low.^[^ ^9^ ^]^ In addition, these conventional treatments are often accompanied by significant adverse effects and a substantial decline in patient quality of life.^[^ ^10^, ^11^, ^12^ ^]^ Consequently, there is an urgent need for the development of novel and more effective therapeutic strategies for GC.

The tumor microenvironment (TME) in GC comprises various immune and stromal components, among which tumor‐associated macrophages (TAMs) play a crucial role.^[^ ^13^, ^14^, ^15^ ^]^ TAMs exhibit a functional dichotomy in tumor biology, capable of both inhibiting and promoting cancer progression. In the context of GC, they have been implicated in promoting tumor proliferation, invasion, and metastasis.^[^ ^16^, ^17^, ^18^ ^]^ Emerging evidence suggests that reprogramming the immune microenvironment—particularly through modulation of TAMs—can markedly improve therapeutic outcomes.^[^ ^19^ ^]^ By altering the polarization or functional state of TAMs, it may be possible to enhance anti‐tumor immune responses and overcome the limitations associated with conventional treatment modalities. Therefore, elucidating the specific roles and regulatory mechanisms of TAMs in GC progression is essential for the development of next‐generation immunotherapeutic strategies.

The interaction between CXCL12 and its receptor CXCR4 plays a pivotal role in regulating cell migration and invasion within the TME.^[^ ^20^, ^21^ ^]^ The CXCL12–CXCR4 signaling axis is known to promote tumor growth and metastasis in various malignancies, including GC.^[^ ^22^, ^23^ ^]^ Beyond directly facilitating tumor cell proliferation and migration, this pathway also modulates the immune landscape of the TME by influencing the activity and phenotype of immune cells, particularly TAMs.^[^ ^24^, ^25^ ^]^ Inhibition of the CXCL12–CXCR4 pathway has been shown to attenuate tumor invasiveness, suppress tumor progression,^[^ ^26^, ^27^, ^28^ ^]^ and reprogram TAMs, thereby initiating potent anti‐tumor immune responses.^[^ ^29^ ^]^ Thus, targeting the CXCL12–CXCR4 axis represents a promising therapeutic strategy for GC.

With recent advances in nanotechnology, nanoparticles (NPs) have emerged as powerful platforms in cancer therapy, offering enhanced drug delivery capabilities with improved targeting specificity and reduced systemic toxicity.^[^ ^30^ ^]^ Engineered NPs serve as multifunctional carriers, capable of increasing drug bioavailability; while, minimizing adverse effects.^[^ ^31^ ^]^ Among them, self‐assembled M2pep‐Cs NPs/Plerixafor NPs represent a next‐generation nanomedicine system designed to specifically inhibit the CXCL12–CXCR4 signaling pathway and reprogram TAMs to potentiate the anti‐tumor immune response. This nanoparticle‐based strategy exemplifies the integration of nanotechnology with immunomodulation and holds the potential to transform conventional GC treatments by offering more effective and safer therapeutic options.

This study aims to explore the application of self‐assembled M2pep‐Cs NPs/Plerixafor NPs in targeting the CXCL12–CXCR4 pathway and reprogramming TAMs to enhance immune‐mediated tumor elimination in GC. The stability and biocompatibility of the NPs are thoroughly evaluated through a series of biophysical and biochemical assessments. Moreover, both in vitro and in vivo models are employed to validate their anti‐tumor efficacy. This research seeks to uncover novel mechanistic insights and establish an innovative therapeutic strategy for GC. If validated in clinical settings, this nanotechnology‐based approach could significantly impact GC treatment paradigms and offer new hope for patients.

The key finding of this study is that M2pep‐Cs NPs/Plerixafor NPs effectively inhibit the CXCL12–CXCR4 pathway, resulting in TAM reprogramming and enhanced immune recognition of GC cells, thereby suppressing tumor growth. This novel immunotherapeutic strategy introduces a new avenue for GC treatment with substantial clinical implications. The manuscript further elucidates the underlying mechanisms of action and explores the potential clinical application of these NPs, providing valuable insights into the development of more effective GC therapies.

## 2 Results

### 2.1 Preparation and Characterization of M2pep‐Cs NPs/Plerixafor NPs

The M2pep‐Cs NPs/Plerixafor NPs developed in this study combine the tumor‐inhibitory effects of Plerixafor with Cs‐based nanotechnology to enhance therapeutic efficacy in GC. By targeting the CXCL12–CXCR4 signaling pathway and reprogramming tumor‐associated M*Φ* within the TME, these NPs aim to improve immune recognition and clearance of tumor cells. This strategy represents a promising immunotherapeutic approach for GC.

**Figure 1:**
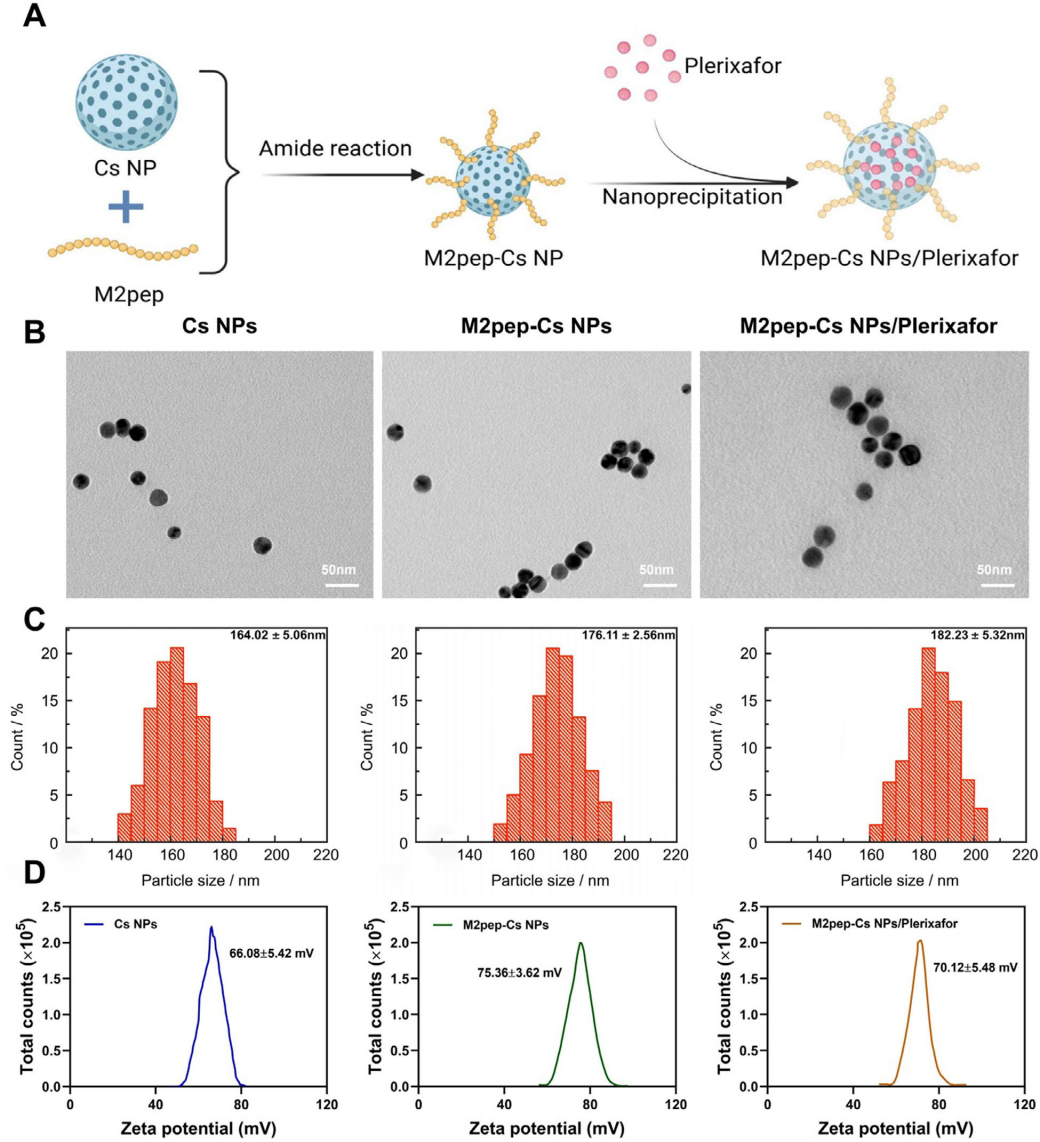
Preparation and characterization of M2pep‐Cs NPs/Plerixafor nanoparticles. Note: A) Schematic diagram of the preparation process for M2pep‐Cs NPs/Plerixafor nanoparticles (Created by BioRender); B) TEM images of Cs NPs, M2pep‐Cs NPs, and M2pep‐Cs NPs/Plerixafor nanoparticles, scale bar: 50 nm; C) average hydrodynamic diameter of Cs NPs, M2pep‐Cs NPs, and M2pep‐Cs NPs/Plerixafor nanoparticles; and D) zeta potential of Cs NPs, M2pep‐Cs NPs, and M2pep‐Cs NPs/Plerixafor nanoparticles. Each experiment was repeated three times, and values are presented as mean ± standard deviation.

M2pep‐Cs NPs/Plerixafor were synthesized as illustrated in **Figure** **1**A. TEM revealed that Cs NPs, M2pep‐Cs NPs, and M2pep‐Cs NPs/Plerixafor were uniformly spherical, densely structured, and well‐dispersed. Notably, M2pep‐Cs NPs/Plerixafor exhibited a smaller average diameter compared to the other NPs (Figure 1B). DLS analysis showed the following characteristics: Cs NPs had a diameter of 164.02 ± 5.06 nm and zeta potential of 66.08 ± 5.42 mV; M2pep‐Cs NPs had a diameter of 176.11 ± 2.56 nm and zeta potential of 75.26 ± 3.62 mV; and M2pep‐Cs NPs/Plerixafor had a diameter of 182.23 ± 5.32 nm and zeta potential of 70.12 ± 5.48 mV. The slight increase in hydrodynamic diameter from Cs NPs to M2pep‐Cs NPs indicated successful surface conjugation of M2pep; while, the further increase in M2pep‐Cs NPs/Plerixafor confirmed effective Plerixafor loading (Figure 1C,D). The discrepancy between TEM and DLS measurements is attributed to the hydration shell and surface‐bound molecules, which are included in DLS but not in TEM.

To confirm M2pep conjugation, Cy5‐labeled M2pep was analyzed using UV–visible absorption and fluorescence emission spectroscopy. Both Cy5‐M2pep‐Cs NPs and Cy5‐M2pep‐Cs NPs/Plerixafor displayed characteristic absorption peaks at 420–480 nm and fluorescence emission peaks at 530–550 nm, confirming successful labeling (Figure S5A, Supporting Information). The stability of M2pep‐Cs NPs/Plerixafor was tested under various physiological conditions (water, PBS, saline, and RPMI‐1640). Only slight size variations were observed, indicating good environmental stability (Figure S5B, Supporting Information). Further, DLS analysis under different pH conditions (5.0, 5.5, 6.5, 7.4, and 8.0) showed minimal fluctuations in particle size and count rate (Figure S5C, Supporting Information), demonstrating that the NPs maintained structural integrity even under mildly acidic conditions such as pH 5.5–6.5. These findings highlight the NPs' favorable kinetic stability and suitability as drug carriers.

To evaluate the pH‐sensitive release profile of Plerixafor, in vitro release studies were conducted at pH 7.4, 6.5, and 5.0. After 2 h, the cumulative release of Plerixafor reached ≈20%, 30%, and 40%, respectively, with a gradual increase over time (Figure S5D, Supporting Information). These results suggest an accelerated release of Plerixafor in the acidic TME, enhancing its therapeutic potential.^[^ ^32^ ^]^

The biocompatibility of the NPs was assessed using CCK‐8 and LIVE/DEAD assays on RAW264.7 cells. The CCK‐8 assay showed comparable proliferation rates among groups with no significant cytotoxicity (Figure S5E, Supporting Information). LIVE/DEAD staining confirmed high cell viability over 3 days of culture, with no discernible differences among groups (Figure S5F,G, Supporting Information). In addition, hemolysis assays revealed minimal hemolytic activity (<5%) across all tested concentrations (Figure S5H, Supporting Information), confirming the NPs’ safety for biomedical applications.

M2 M*Φ*, which exhibits tumor‐promoting phenotypes, plays a critical role in the progression of GC. These cells secrete pro‐inflammatory and pro‐angiogenic factors that enhance tumor cell proliferation, invasion, and metastasis.^[^ ^33^ ^]^ Further, M2 M*Φ* contributes to the establishment of an immunosuppressive TME, thereby attenuating anti‐tumor immune responses and facilitating disease advancement.^[^ ^34^ ^]^ To assess the targeting efficiency of M2pep‐Cs NPs/Plerixafor NPs toward M*Φ* subtypes, THP‐1 monocytes were differentiated into macrophages using PMA and further polarized into M2 M*Φ* through treatment with IL‐4 and IL‐13. Successful polarization was confirmed by immunophenotyping, which revealed decreased expression of CD86 and increased expression of CD206 in M2 M*Φ* relative to unpolarized M*Φ* (Figure S6A, Supporting Information). Uptake studies were conducted using flow cytometry to quantify the internalization of Plerixafor and nanoparticle formulations. M2pep‐Cs NPs/Plerixafor exhibited significantly greater cellular uptake compared to free Plerixafor, with uptake levels reaching ≈33.7% in M*Φ* and 83.8% in M2 M*Φ*, versus 6.7% and 13.4%, respectively, for free Plerixafor (Figure S6B, Supporting Information). In addition, immunofluorescence microscopy further corroborated the flow cytometry results, visualizing both the intrinsic fluorescence of Plerixafor and the Cy5‐labeled M2pep. Fluorescence intensity in the M2pep‐Cs NPs/Plerixafor group was markedly higher than that of Plerixafor alone, especially in M2 M*Φ* compared to unpolarized M*Φ*. However, Cy5 fluorescence did not differ significantly between M2pep‐Cs NPs and M2pep‐Cs NPs/Plerixafor, indicating that Plerixafor loading did not interfere with M2pep‐mediated targeting (Figure S6C, Supporting Information).

Collectively, these results demonstrate that M2pep‐Cs NPs/Plerixafor NPs exhibit excellent in vitro stability, sustained release properties, and efficient, selective uptake by M2 macrophages—highlighting their potential as an immune‐targeted therapeutic strategy for GC.

### 2.2 Multi‐Omics Identification of Key Pathways Targeted by M2pep‐Cs NPs/Plerixafor in GC Suppression

Elucidating the molecular mechanisms underlying the anti‐tumor effects of M2pep‐Cs NPs/Plerixafor is essential for optimizing therapeutic strategies against GC. A GC mouse model was first established via MFC cell injection. In vivo imaging demonstrated that tumor growth in the GC group was markedly accelerated compared to the normal control group. In contrast, mice treated with M2pep‐Cs NPs/Plerixafor exhibited a significantly reduced tumor growth rate (Figure S7A, Supporting Information). Histological evaluation via H&E staining revealed extensive tumor cell infiltration in the GC group, which was markedly attenuated in the M2pep‐Cs NPs/Plerixafor‐treated group (Figure S7B, Supporting Information). IHC analysis of tumor tissues further showed a significant reduction in Ki67‐positive proliferating cells following nanoparticle treatment compared to the untreated GC group (Figure S7C, Supporting Information). TUNEL staining demonstrated a significant increase in apoptotic cells in tumors from the M2pep‐Cs NPs/Plerixafor group relative to the GC group (Figure S7D, Supporting Information).

To assess biodistribution, Cy5‐labeled M2pep‐Cs NPs/Plerixafor was administered via tail vein injection into tumor‐bearing BALB/c mice. In vivo fluorescence imaging, followed by ex vivo imaging of major organs, revealed substantial nanoparticle accumulation in tumor tissues, with minimal off‐target distribution to organs such as the heart, liver, spleen, lungs, and kidneys (Figure S7E, Supporting Information).

**Figure 2:**
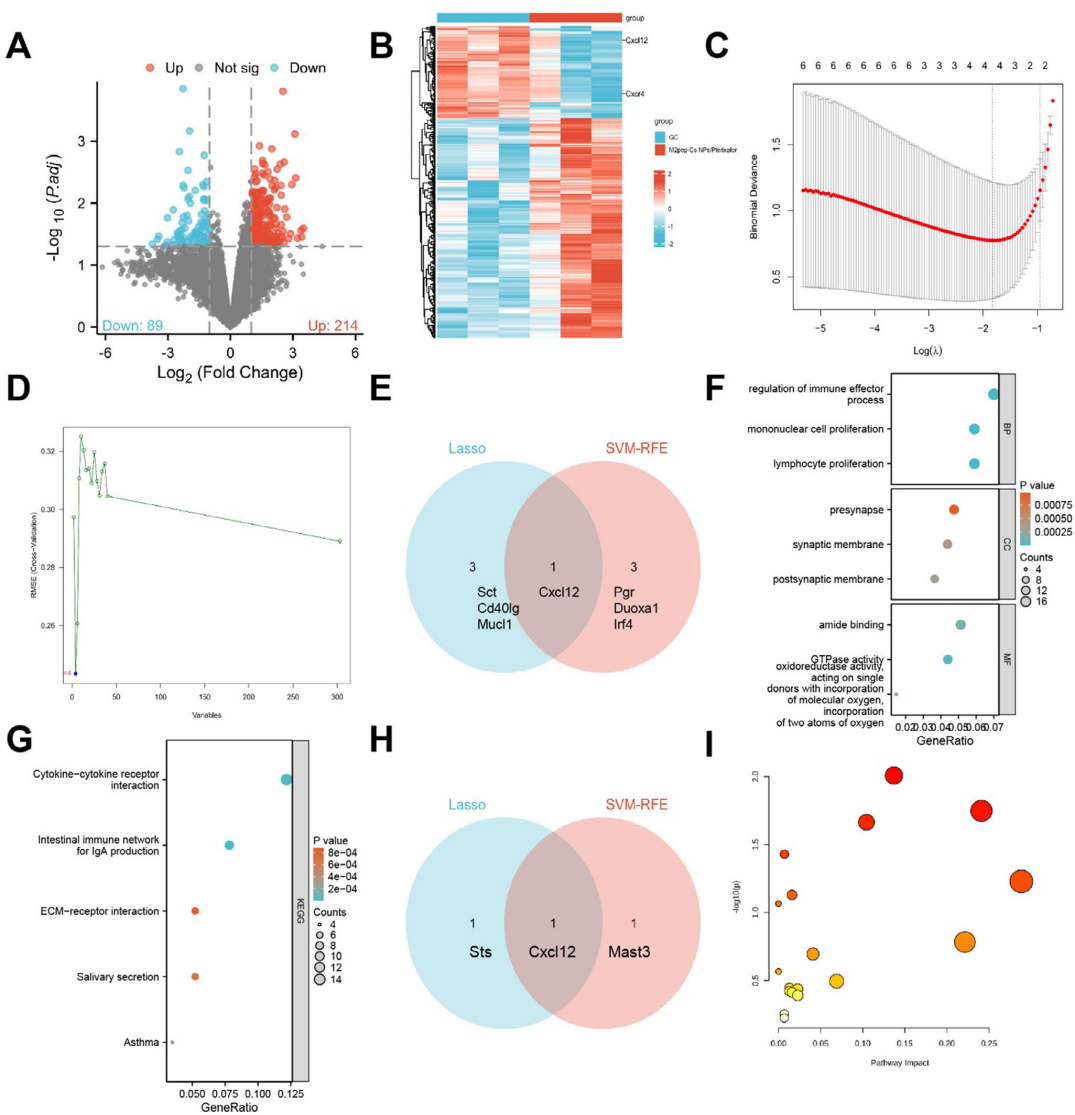
Potential pathways for inhibiting GC identified through multi‐omics data analysis of M2pep‐Cs NPs/Plerixafor nanoparticles. Note: A) Volcano plot of differentially expressed genes between GC and M2pep‐Cs NPs/Plerixafor groups based on high‐throughput sequencing data from 3 GC and 3 M2pep‐Cs NPs/Plerixafor mouse tumor tissues; B) heatmap of differentially expressed genes between GC and M2pep‐Cs NPs/Plerixafor groups based on high‐throughput sequencing data from 3 GC and 3 M2pep‐Cs NPs/Plerixafor mouse tumor tissues; C) LASSO algorithm selected nine feature genes; D) SVM‐RFE algorithm selected ten feature genes; E) Venn diagram showing the intersection of 1 gene identified by both machine learning methods; F) GO enrichment bubble chart for differentially expressed genes between GC and M2pep‐Cs NPs/Plerixafor groups based on high‐throughput sequencing data from 3 GC and 3 M2pep‐Cs NPs/Plerixafor mouse tumor tissues; G) KEGG enrichment bubble chart for differentially expressed genes between GC and M2pep‐Cs NPs/Plerixafor groups based on high‐throughput sequencing data from 3 GC and 3 M2pep‐Cs NPs/Plerixafor mouse tumor tissues; H) Venn diagram of 1 protein identified by intersecting results from two machine learning methods based on proteomics sequencing data; and I) KEGG pathway analysis of 27 differentially expressed metabolites based on metabolomics data.

High‐throughput RNA sequencing of tumor tissues revealed pronounced gene expression differences between the M2pep‐Cs NPs/Plerixafor group and the GC control group. Specifically, 214 genes were significantly upregulated, and 89 genes were downregulated following treatment (**Figure** **2**A,B). To further refine the analysis, two machine learning algorithms—Lasso regression and SVM‐RFE—were employed to identify key feature genes. The Lasso model identified Sct, Cd40lg, Mucl1, and Cxcl12 as pivotal genes (Figure 2C); while, the SVM‐RFE algorithm selected Duoxa1, Pgr, Cxcl12, and Irf4 (Figure 2D). Cxcl12 emerged as the common feature gene identified by both algorithms, as illustrated by the intersection in the Venn diagram (Figure 2E).

GO enrichment analysis revealed that the differentially expressed genes were predominantly involved in biological processes (BP) such as immune effector regulation, lymphocyte proliferation, mononuclear cell proliferation, and B cell proliferation. Cellular component (CC) analysis showed enrichment in the postsynaptic membrane, synaptic membrane, presynapse, and collagen trimer structures. In terms of molecular functions (MF), the genes were enriched for GTPase activity, amide binding, oxidoreductase activity (acting on single donors with incorporation of molecular oxygen), and mannose binding (Figure 2F). Pathway enrichment based on the KEGG revealed that these genes were significantly associated with immune‐related pathways, including the intestinal immune network for IgA production, cytokine–cytokine receptor interaction, asthma, and salivary secretion (Figure 2G).

Machine learning techniques were instrumental in identifying CXCL12, the principal ligand of CXCR4 (also known as stromal cell‐derived factor 1 or SDF‐1).^[^ ^35^ ^]^ The CXCL12–CXCR4 axis plays a pivotal role in diverse physiological and pathological processes, including immune regulation, hematopoiesis, inflammation, and cancer metastasis.^[^ ^36^ ^]^ GO and KEGG enrichment analyses further supported the immune relevance of the differential genes identified in this study.

To explore the molecular pathways by which M2pep‐Cs NPs/Plerixafor inhibit GC growth, tumor tissues from three mice in each of the GC and treatment groups were analyzed. Principal component analysis (PCA) and loading plots revealed clear group separation (Figure S8A–C, Supporting Information). OPLS‐DA confirmed robust model discrimination with strong explanatory power (*R^2^Y* = 0.996 > 0.8) (Figure S8D–F, Supporting Information). Proteomic analysis identified 278 differentially expressed proteins between groups, including 21 upregulated and 24 downregulated proteins (Figure S9A,B, Supporting Information). Feature protein selection was further refined using Lasso regression and SVM‐RFE algorithms. Lasso identified Cxcl12 and Sts as characteristic proteins (Figure S9C, Supporting Information); while, SVM‐RFE identified Mast3 and Cxcl12 (Figure S9D, Supporting Information). The intersection of these results again confirmed CXCL12 as a shared key protein, illustrated via a Venn diagram (Figure 2H).

GO enrichment of these proteins revealed BP involvement in mitochondrial translation, response to granulocyte M*Φ*colony‐stimulating factor, and multi‐organism reproductive processes. MF enrichment included sulfuric ester hydrolase activity, ubiquitin‐protein transferase activity, and acetylcholine receptor binding (Figure S9E,F, Supporting Information). These findings align with transcriptomic data, reinforcing the critical role of immune pathways.

For metabolomic profiling, tumor tissues from six mice per group were analyzed. PCA and loading plots again demonstrated distinct metabolic signatures between the GC and M2pep‐Cs NPs/Plerixafor groups (Figure S10A–C, Supporting Information), further validated by OPLS‐DA (*R^2^Y* = 0.944) (Figure S10D–F, Supporting Information). KEGG‐based pathway enrichment of 27 differentially expressed metabolites revealed significant involvement in arginine biosynthesis, pentose and glucuronate interconversions, beta‐alanine metabolism, and glutathione metabolism (Figure 2I; Table S2, Supporting Information).

To extend these findings to clinical relevance, we analyzed CXCL12 and CXCR4 expression using the TIMER database. Both genes were highly expressed across multiple tumor types, including GC (Figure S11A,B, Supporting Information). Correlation analysis revealed a strong association between CXCL12 and macrophages and between CXCR4 and dendritic cells in the GC TME (Figure S11C,D, Supporting Information). Survival analysis stratified by CXCL12 and CXCR4 expression indicated that high CXCL12 expression was associated with reduced patient survival, and both genes significantly influenced macrophage infiltration (Figure S11E,F, Supporting Information). Further subtype‐specific analysis using the TISIDB database revealed consistently high expression levels of CXCL12 and CXCR4 across all GC subtypes (Figure S12A,B,D,E, Supporting Information). Immune cell correlation analysis confirmed significant associations between CXCL12 and M*Φ*, effector memory CD8^+^ and CD4^+^ T cells, and NKT cells. Similarly, CXCR4 expression correlated with activated CD8^+^ T cells, effector memory T cells, and M*Φ* (Figure S12C,E,F, Supporting Information).

Collectively, this multimodal omics analysis highlights CXCL12 as a key immunoregulatory target modulated by M2pep‐Cs NPs/Plerixafor. Integration of transcriptomic, proteomic, and metabolomic data supports the hypothesis that these NPs suppress GC growth by disrupting the CXCL12–CXCR4 axis and remodeling immune cell interactions within the TME. These findings provide a strong mechanistic rationale for targeting CXCL12 in GC immunotherapy and offer a theoretical foundation for advancing precision nanomedicine in oncology.

### 2.3 M2pep‐Cs NPs/Plerixafor NPs Inhibit the CXCL12–CXCR4 Signaling Pathway, Affecting TAM Reprogramming to Enhance Immune Recognition and Reduce Proliferation of GC Cells

**Figure 3:**
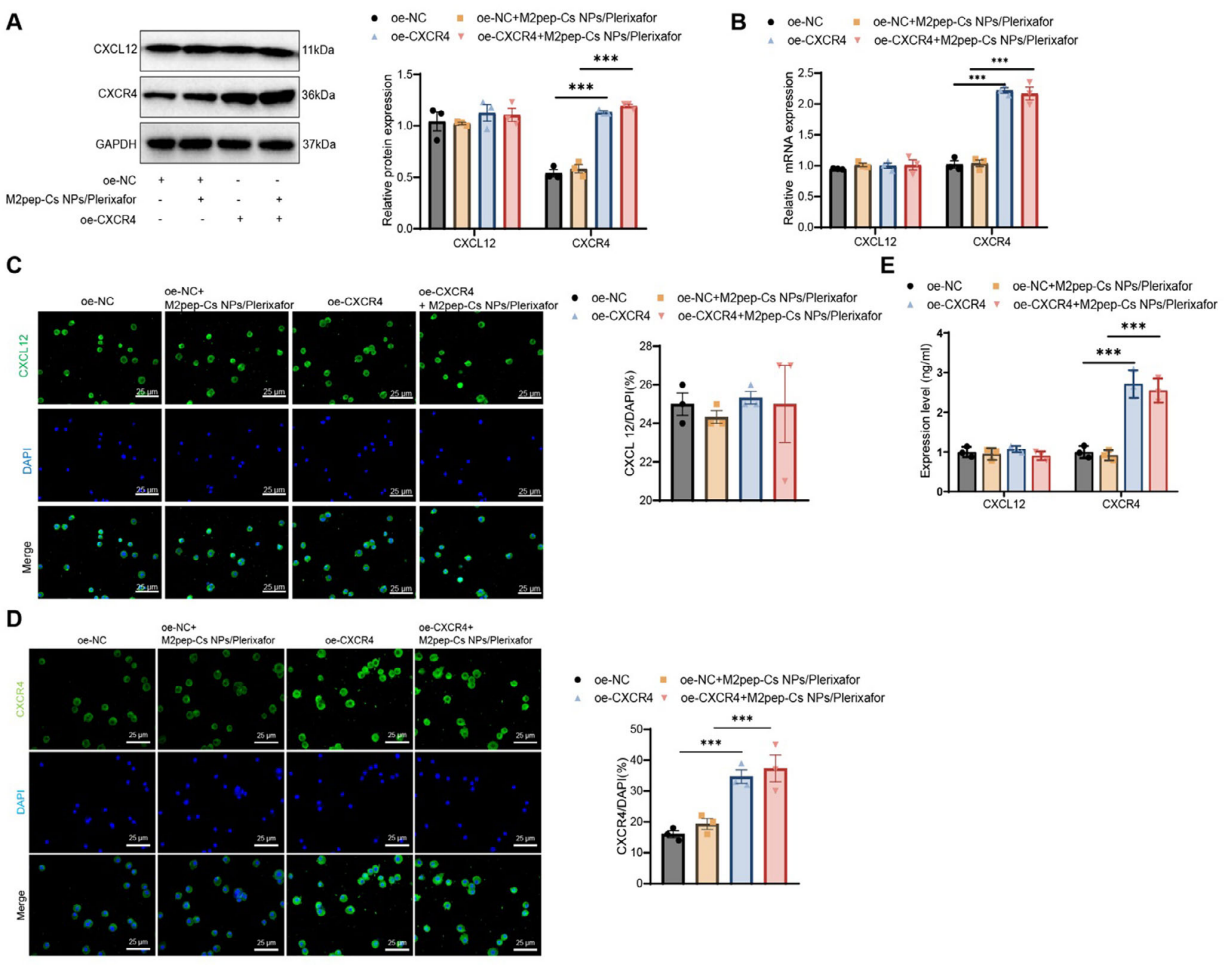
Effect of M2pep‐Cs NPs/Plerixafor nanoparticles on the CXCL12–CXCR4 signaling pathway. Note: A) Western blot analysis of CXCL12 and CXCR4 protein expression levels in RAW264.7 cells (Created by BioRender); B) RT‐qPCR analysis of CXCL12 and CXCR4 mRNA expression levels in RAW264.7 cells; C) immunofluorescence staining of CXCL12 expression in RAW264.7 cells, scale bar: 25 µm; D) immunofluorescence staining of CXCR4 expression in RAW264.7 cells, scale bar: 25 µm; and E) ELISA analysis of CXCL12 and CXCR4 protein levels in RAW264.7 cell culture supernatants. Each experiment was repeated three times, and values are presented as mean ± standard deviation. Multiple group comparison using one‐way ANOVA, ***p < 0.001.

Recent research has increasingly emphasized the critical role of TAMs within the TME in promoting tumor progression and modulating therapeutic outcomes. In particular, the CXCL12–CXCR4 signaling pathway has been implicated in regulating TAM polarization and function. This study evaluated how M2pep‐Cs NPs/Plerixafor NPs influence this pathway and its subsequent effects on the polarization of macrophages and the behavior of GC cells. Initially, Western blot results demonstrated that compared with the oe‐NC group, the expression levels of CXCL12 and CXCR4 proteins remained unchanged in the oe‐NC+M2pep‐Cs NPs/Plerixafor group. In the oe‐CXCR4 group, CXCR4 protein levels were significantly upregulated; while, CXCL12 expression remained unchanged. Similarly, in the oe‐CXCR4+M2pep‐Cs NPs/Plerixafor group, CXCR4 expression was further increased compared to the oe‐NC+M2pep‐Cs NPs/Plerixafor group, whereas CXCL12 levels remained unaffected (**Figure** **3**A). RT‐qPCR analysis supported these findings. Compared with the oe‐NC group, no significant changes in CXCL12 or CXCR4 mRNA expression were observed in the oe‐NC+M2pep‐Cs NPs/Plerixafor group. However, CXCR4 mRNA expression was significantly elevated in the oe‐CXCR4 group, with no alteration in CXCL12 mRNA levels. Compared to the oe‐NC+M2pep‐Cs NPs/Plerixafor group, CXCR4 mRNA expression was significantly upregulated in the oe‐CXCR4+M2pep‐Cs NPs/Plerixafor group, whereas CXCL12 mRNA expression remained unchanged (Figure 3B). Immunofluorescence staining further confirmed these molecular findings. In the oe‐NC+M2pep‐Cs NPs/Plerixafor group, CXCL12 and CXCR4 fluorescence intensities were unchanged relative to the oe‐NC group. In contrast, the oe‐CXCR4 and oe‐CXCR4+M2pep‐Cs NPs/Plerixafor groups showed significantly increased CXCR4 fluorescence, with CXCL12 fluorescence remaining stable (Figure 3C,D). ELISA assays of RAW264.7 cell culture supernatants corroborated these trends. No changes in CXCL12 or CXCR4 secretion were observed in the oe‐NC+M2pep‐Cs NPs/Plerixafor group compared to the oe‐NC group. However, both the oe‐CXCR4 and oe‐CXCR4+M2pep‐Cs NPs/Plerixafor groups exhibited elevated CXCR4 protein levels; while, CXCL12 remained unchanged (Figure 3E).

**Figure 4:**
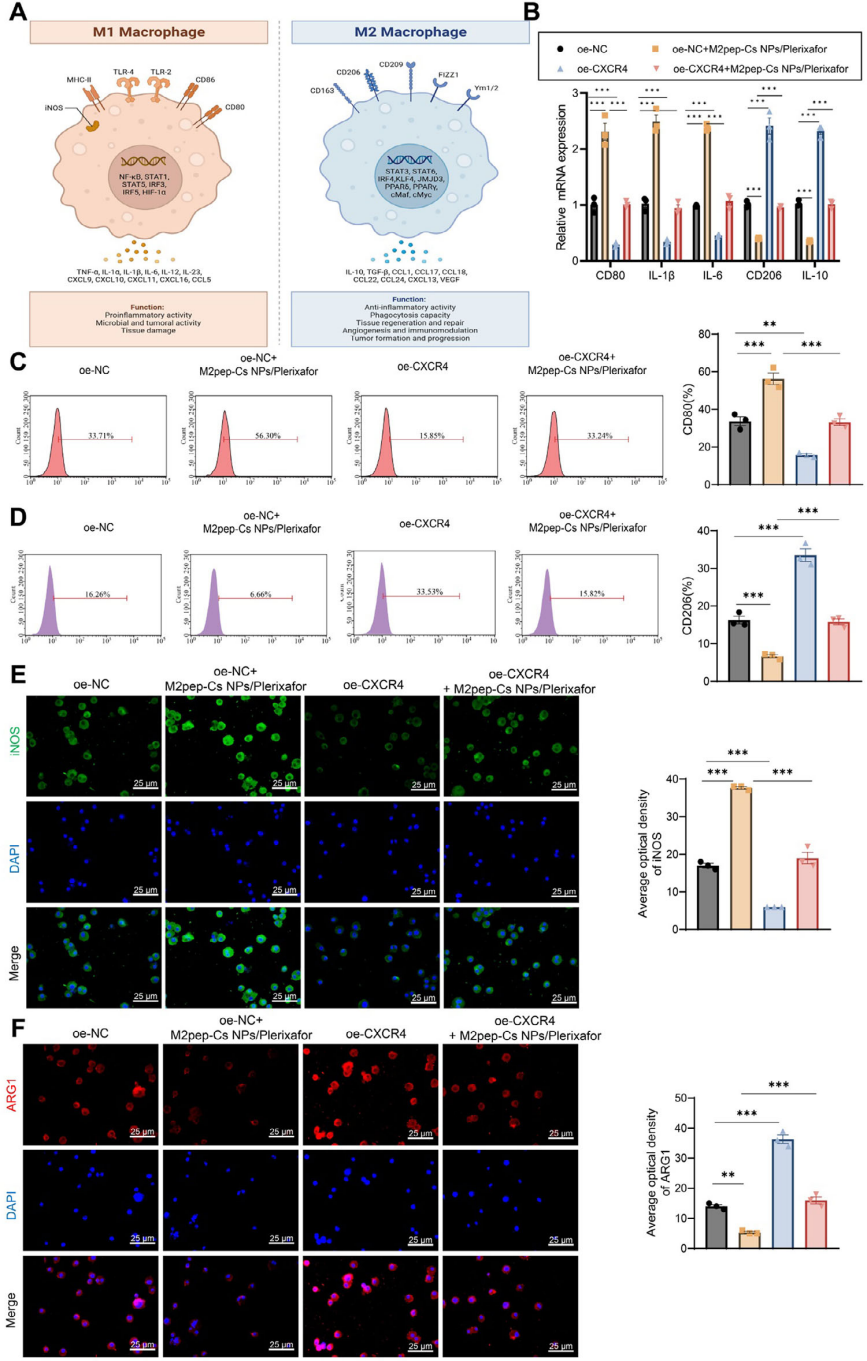
Impact of M2pep‐Cs NPs/Plerixafor nanoparticles on TAM polarization via the CXCL12–CXCR4 signaling pathway. Note: A) Schematic representation of TAM polarization (Created by BioRender); B) RT‐qPCR analysis of mRNA levels of CD80, IL‐1β, IL‐6, CD206, and IL‐10 in RAW264.7 cells in different groups; C) flow cytometry analysis of CD80 expression in RAW264.7 cells in different groups; D) flow cytometry analysis of CD206 expression in RAW264.7 cells in different groups; E) immunofluorescence detection of iNOS protein expression in RAW264.7 cells in different groups, scale bar: 25 µm; and F) immunofluorescence detection of ARG1 protein expression in RAW264.7 cells in different groups, scale bar: 25 µm. Each experiment was repeated three times, and values are presented as mean ± standard deviation. Multiple group comparison using one‐way ANOVA, **p < 0.01, and ***p < 0.001.

To further investigate the role of CXCL12–CXCR4 signaling in TAM polarization, RAW264.7 macrophages were employed. The experimental design is illustrated in **Figure** **4**A. RT‐qPCR analysis revealed that compared to the oe‐NC group, the oe‐NC+M2pep‐Cs NPs/Plerixafor group showed significant upregulation of M1 TAM cytokines CD80, IL‐1β, and IL‐6 mRNA levels, and a significant downregulation of M2 TAM cytokines CD206 and IL‐10 mRNA levels. In contrast, the oe‐CXCR4 group displayed a significant downregulation of CD80, IL‐1β, and IL‐6 mRNA levels and an upregulation of CD206 and IL‐10. Relative to the oe‐NC+M2pep‐Cs NPs/Plerixafor group, the oe‐CXCR4+M2pep‐Cs NPs/Plerixafor group exhibited further downregulation of CD80, IL‐1β, and IL‐6 mRNA levels and upregulation of CD206 and IL‐10 mRNA levels (Figure 4B).

Flow cytometry results demonstrated that compared to the oe‐NC group, there was a significant increase in the proportion of CD80 in M1‐type TAMs in the oe‐NC+M2pep‐Cs NPs/Plerixafor group; while, the oe‐CXCR4 group showed a significant decrease. The oe‐CXCR4+M2pep‐Cs NPs/Plerixafor group showed a significant decrease in CD80 compared to the oe‐NC+M2pep‐Cs NPs/Plerixafor group (Figure 4C). Regarding M2 TAM markers, CD206 significantly decreased in the oe‐NC+M2pep‐Cs NPs/Plerixafor group but increased in the oe‐CXCR4 group. This increase was more pronounced in the oe‐CXCR4+M2pep‐Cs NPs/Plerixafor group compared to the oe‐NC+M2pep‐Cs NPs/Plerixafor group (Figure 4D). Immunofluorescence results showed that iNOS protein expression was significantly increased in the oe‐NC+M2pep‐Cs NPs/Plerixafor group compared to the oe‐NC group but significantly decreased in the oe‐CXCR4+M2pep‐Cs NPs/Plerixafor group relative to the oe‐NC+M2pep‐Cs NPs/Plerixafor group (Figure 4E). Compared to the oe‐NC group, ARG1 protein expression was significantly downregulated in the oe‐NC+M2pep‐Cs NPs/Plerixafor group; while, in the oe‐CXCR4 group, ARG1 protein expression was significantly upregulated. Compared to the oe‐NC+M2pep‐Cs NPs/Plerixafor group, ARG1 protein expression was significantly increased in the oe‐CXCR4+M2pep‐Cs NPs/Plerixafor group (Figure 4F).

**Figure 5:**
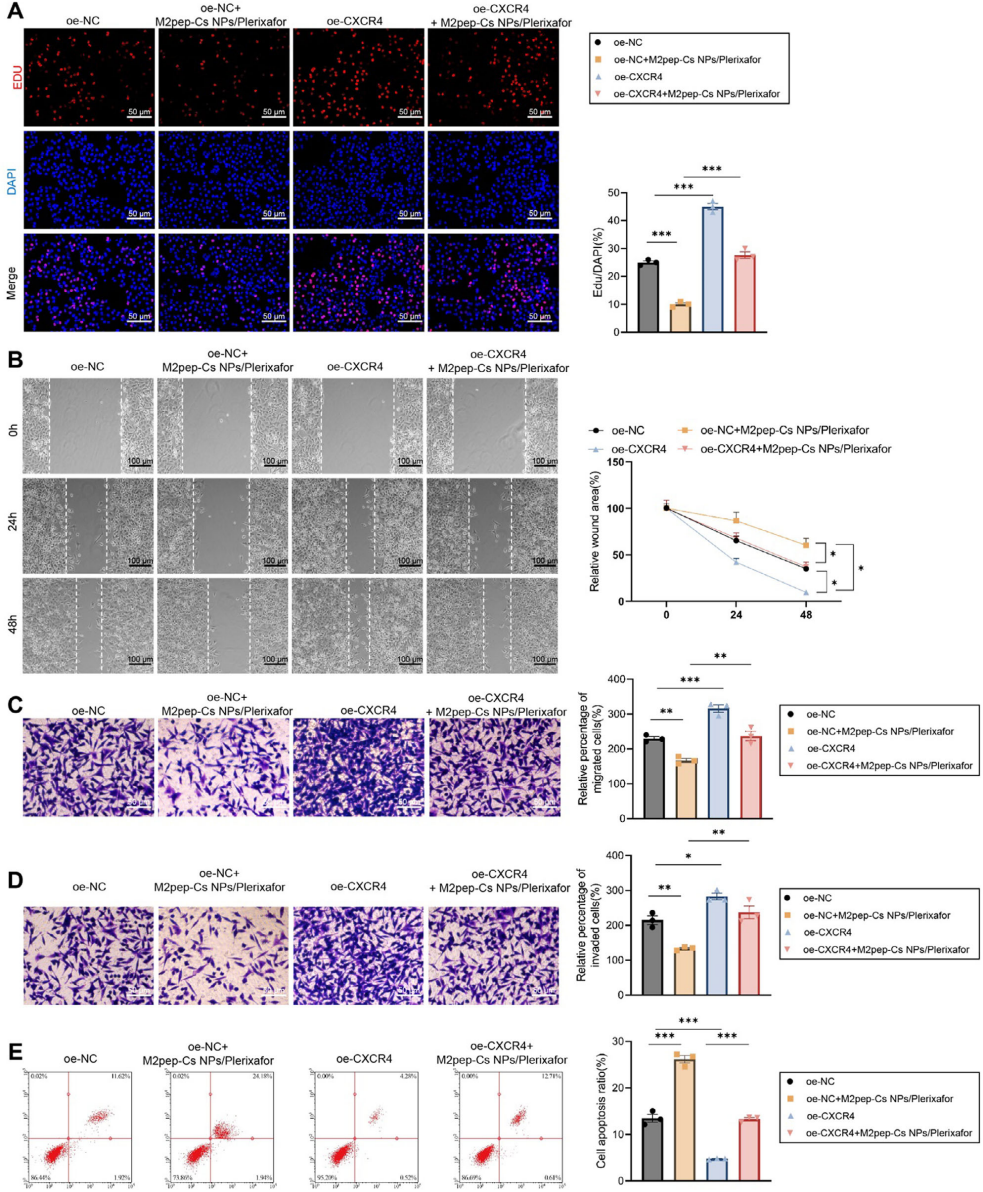
M2pep‐Cs NPs/Plerixafor nanoparticles inhibit the CXCL12–CXCR4 signaling pathway, reprogramming TAMs and enhancing the immune system's response to GC cells. Note: A) Edu immunofluorescence staining to assess MFC cell proliferation, scale bar: 50 µm; B) wound healing assay to measure the migration speed of MFC cells, scale bar: 100 µm; C) Transwell assay to evaluate MFC cell migration, scale bar: 50 µm; D) Transwell assay to assess MFC cell invasion, scale bar: 50 µm; and E) flow cytometry analysis of MFC cell apoptosis. Each experiment was repeated three times, and values are presented as mean ± standard deviation. Multiple group comparisons were conducted using one‐way ANOVA; while, data from different time points were analyzed using two‐way ANOVA, *p < 0.05, **p < 0.01, and ***p < 0.001.

EdU fluorescence assays revealed significant reductions in cell proliferation in the oe‐NC+M2pep‐Cs NPs/Plerixafor group compared to the oe‐NC group, whereas the oe‐CXCR4 group showed an increase. A notable increase in proliferation was observed in the oe‐CXCR4+M2pep‐Cs NPs/Plerixafor group compared to the oe‐NC+M2pep‐Cs NPs/Plerixafor group (**Figure** **5**A). Scratch assays indicated a marked decrease in wound closure speed in the oe‐NC+M2pep‐Cs NPs/Plerixafor group compared to the oe‐NC group, which contrasted with the increased healing speed in the oe‐CXCR4 group. Compared to the oe‐NC+M2pep‐Cs NPs/Plerixafor group, the oe‐CXCR4+M2pep‐Cs NPs/Plerixafor group exhibited a significantly faster wound closure (Figure 5B). Transwell assays revealed that migration and invasion were significantly reduced in the oe‐NC+M2pep‐Cs NPs/Plerixafor group relative to the oe‐NC group; while, the oe‐CXCR4 group showed increases. These capabilities were further enhanced in the oe‐CXCR4+M2pep‐Cs NPs/Plerixafor group compared to the oe‐NC+M2pep‐Cs NPs/Plerixafor group (Figure 5C,D). Flow cytometry revealed that apoptosis was significantly increased in the oe‐NC+M2pep‐Cs NPs/Plerixafor group compared to the oe‐NC group, decreased in the oe‐CXCR4 group, and further reduced in the oe‐CXCR4+M2pep‐Cs NPs/Plerixafor group compared to the oe‐NC+M2pep‐Cs NPs/Plerixafor group (Figure 5E). Collectively, these results demonstrate that M2pep‐Cs NPs/Plerixafor NPs effectively reprogrammed TAMs from a tumor‐promoting M2 phenotype to a tumor‐suppressive M1 phenotype. This phenotypic shift led to reduced proliferation, invasion, and migration of GC cells, alongside increased apoptosis. These findings support the use of M2pep‐Cs NPs/Plerixafor as a promising immunotherapeutic strategy for the treatment of GC.

### 2.4 Inhibition of the CXCL12–CXCR4 Signaling Pathway by M2pep‐Cs NPs/Plerixafor NPs Enhances Immune Recognition and Suppresses Tumor Growth in a GC Mouse Model

**Figure 6:**
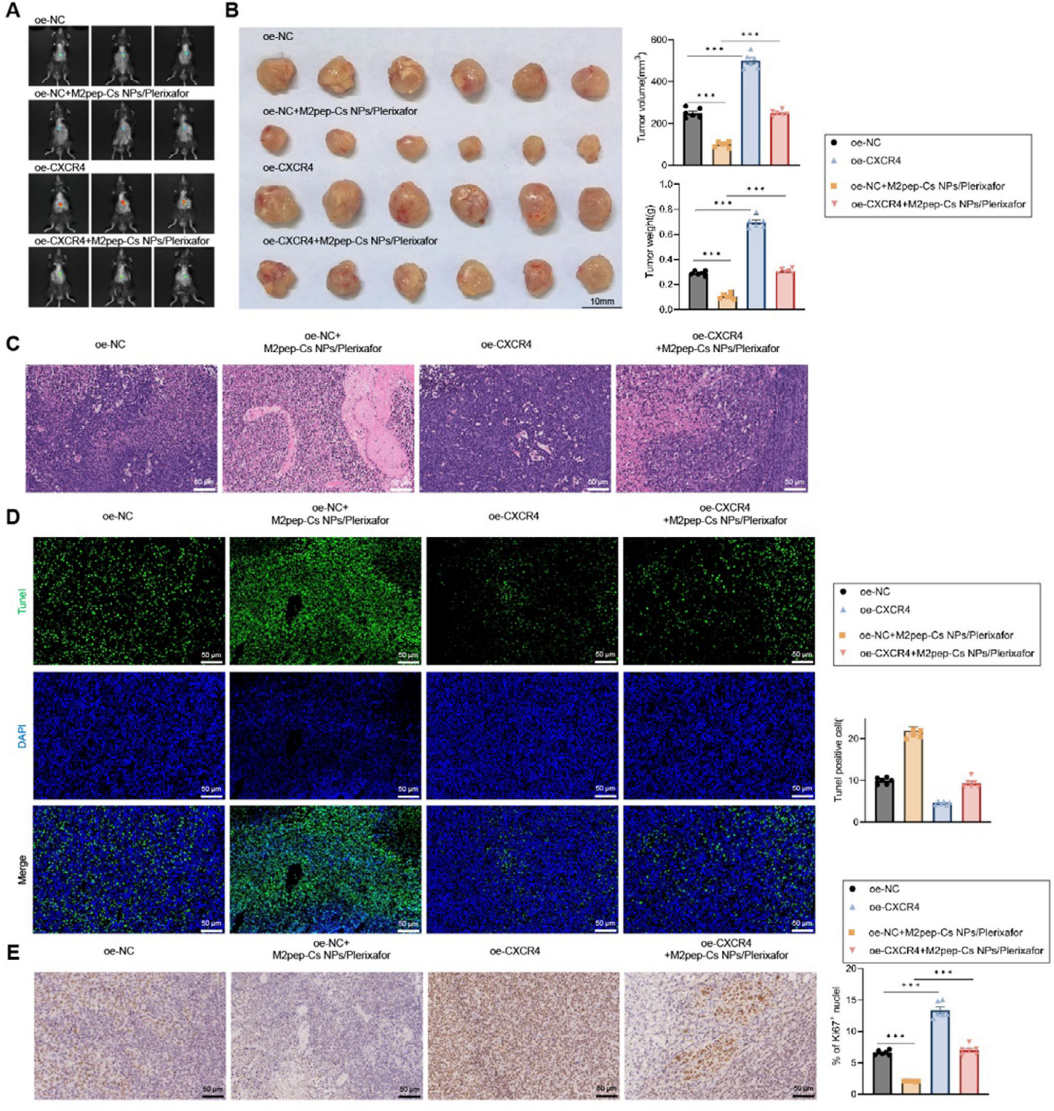
M2pep‐Cs NPs/Plerixafor nanoparticles inhibit tumor growth in GC mouse models. Note: A) In vivo imaging of tumor growth in different groups of mice; B) tumor images and measurements of tumor volume and weight in each group of mice; C) H&E staining of tumor tissue morphology and structure in each group, scale bar: 50 µm; D) TUNEL staining to assess apoptosis in tumor tissues of each group, scale bar: 50 µm; and E) immunohistochemical analysis of Ki67 expression in tumor tissues of each group, scale bar: 50 µm. Each group consisted of six mice, and values are presented as mean ± standard deviation. Multiple group comparisons were conducted using one‐way ANOVA, ***p < 0.001.

To investigate the immunomodulatory and anti‐tumor effects of M2pep‐Cs NPs/Plerixafor NPs in vivo, a murine GC model was established by intraperitoneal injection of MFC cells. This model was used to assess the influence of nanoparticle‐mediated inhibition of the CXCL12–CXCR4 signaling pathway on TAM reprogramming and tumor progression. Initially, in vivo imaging demonstrated that compared to the oe‐NC group, tumor growth in the oe‐NC+M2pep‐Cs NPs/Plerixafor group was significantly reduced; while, the oe‐CXCR4 group showed increased tumor growth rates. Further, tumor growth rates significantly accelerated in the oe‐CXCR4+M2pep‐Cs NPs/Plerixafor group relative to the oe‐NC+M2pep‐Cs NPs/Plerixafor group (**Figure** **6**A). A gross examination of tumor morphology, along with quantitative assessments of tumor volume and weight, confirmed these findings. Tumor size and mass were significantly reduced in the oe‐NC+M2pep‐Cs NPs/Plerixafor group compared to the oe‐NC group. In contrast, both were significantly increased in the oe‐CXCR4 group, with further increases in the oe‐CXCR4+M2pep‐Cs NPs/Plerixafor group (Figure 6B). Histological assessments using H&E staining showed that cellular infiltration in tumor tissues was significantly reduced in the oe‐NC+M2pep‐Cs NPs/Plerixafor group compared to the oe‐NC group, whereas infiltration was significantly increased in the oe‐CXCR4 group and even more so in the oe‐CXCR4+M2pep‐Cs NPs/Plerixafor group (Figure 6C). TUNEL staining revealed that tumor cell apoptosis was significantly higher in the oe‐NC+M2pep‐Cs NPs/Plerixafor group than in the oe‐NC group. Conversely, apoptosis was significantly reduced in the oe‐CXCR4 group and further decreased in the M2pep‐Cs NPs/Plerixafor+oe‐CXCR4 group compared to the oe‐NC+M2pep‐Cs NPs/Plerixafor group (Figure 6D). Immunohistochemical analysis showed that the expression of the proliferation marker Ki67^+^ was significantly decreased in the oe‐NC+M2pep‐Cs NPs/Plerixafor group compared to the oe‐NC group. On the contrary, Ki67^+^ expression was significantly increased in the oe‐CXCR4 group and further increased in the M2pep‐Cs NPs/Plerixafor+oe‐CXCR4 group (Figure 6E). Together, these results demonstrate that M2pep‐Cs NPs/Plerixafor NPs effectively inhibit tumor growth in vivo by modulating the CXCL12–CXCR4 axis and promoting TAM reprogramming. Notably, overexpression of CXCR4 was sufficient to reverse the immune‐activating and tumor‐inhibitory effects of the NPs.

**Figure 7:**
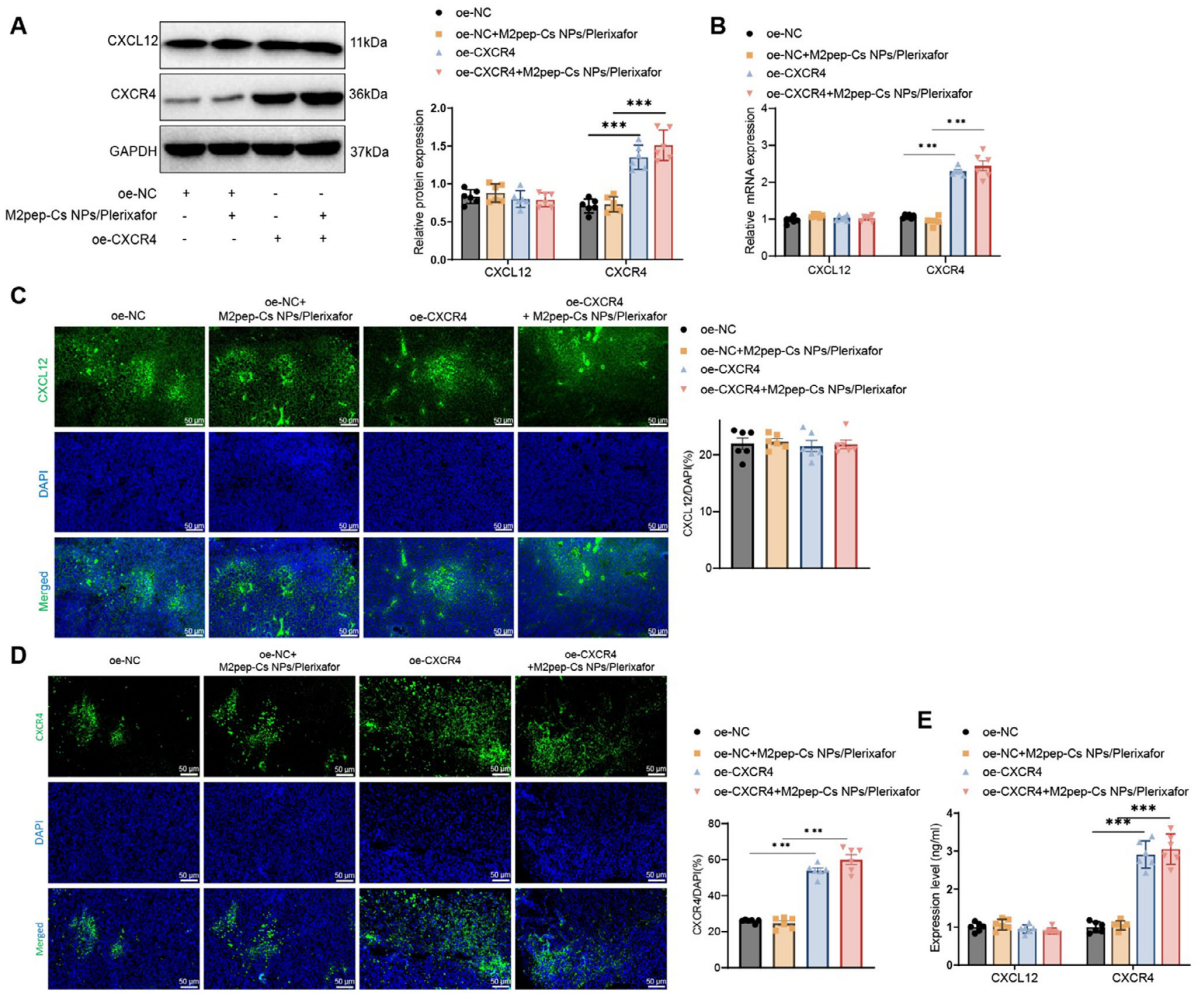
Impact of M2pep‐Cs NPs/Plerixafor nanoparticles on the CXCL12–CXCR4 signaling pathway. Note: A) Western blot analysis of CXCL12 and CXCR4 protein expression levels in mouse tumor tissues; B) RT‐PCR analysis of CXCL12 and CXCR4 mRNA expression levels in mouse tumor tissues; C) immunofluorescence staining of CXCL12 expression in mouse tumor tissues, scale bar: 50 µm; D) immunofluorescence staining of CXCR4 expression in mouse tumor tissues, scale bar: 50 µm; and E) ELISA analysis of CXCL12 and CXCR4 protein levels in tumor tissues. Each group consisted of six mice, and values are presented as mean ± standard deviation. Multiple group comparisons were conducted using one‐way ANOVA, ***p < 0.001.

Initial Western blot results demonstrated a significant decrease in CXCR4 protein expression in the tumor tissues of mice treated with oe‐NC+M2pep‐Cs NPs/Plerixafor compared to the oe‐NC group; while, CXCL12 protein levels remained unchanged. Conversely, in the oe‐CXCR4 group, CXCR4 protein expression was significantly increased with no change in CXCL12 levels. Compared to the oe‐NC+M2pep‐Cs NPs/Plerixafor group, the oe‐CXCR4+M2pep‐Cs NPs/Plerixafor group showed a significant increase in CXCR4 protein expression without any alteration in CXCL12 protein levels (**Figure** **7**A). RT‐qPCR results showed that compared to the oe‐NC group, the mRNA expression levels of CXCR4 and CXCL12 in the tumor tissues of the oe‐NC+M2pep‐Cs NPs/Plerixafor group remained unchanged; while, CXCR4 mRNA expression was significantly upregulated in the tumor tissues of the oe‐CXCR4 group, with no change in CXCL12 mRNA expression. Compared to the oe‐NC+M2pep‐Cs NPs/Plerixafor group, CXCR4 mRNA expression in the tumor tissues of the oe‐CXCR4+M2pep‐Cs NPs/Plerixafor group was significantly upregulated; while, CXCL12 mRNA expression remained unchanged (Figure 7B). The trends in the Western blot results were consistent with those observed in the RT‐qPCR results. Immunofluorescence analysis showed that compared to the oe‐NC group, the fluorescence intensity of CXCR4 and CXCL12 in the tumor tissues of the oe‐NC+M2pep‐Cs NPs/Plerixafor group remained unchanged; while, CXCR4 fluorescence intensity was significantly increased in the tumor tissues of the oe‐CXCR4 group, with no change in CXCL12 fluorescence intensity. Compared to the oe‐NC+M2pep‐Cs NPs/Plerixafor group, the fluorescence intensity of CXCR4 in the tumor tissues of the oe‐CXCR4+M2pep‐Cs NPs/Plerixafor group was significantly increased; while, CXCL12 fluorescence intensity remained unchanged (Figure 7C,D). The results showed that compared to the oe‐NC group, the CXCR4 and CXCL12 protein levels remained unchanged in the tumor tissues of the oe‐NC+M2pep‐Cs NPs/Plerixafor group. However, in the oe‐CXCR4 group, CXCR4 protein expression was significantly upregulated; while, CXCL12 expression remained unchanged. Compared to the oe‐NC+M2pep‐Cs NPs/Plerixafor group, the oe‐CXCR4+M2pep‐Cs NPs/Plerixafor group exhibited significantly increased CXCR4 protein expression; while, CXCL12 expression remained unchanged (Figure 7E).

**Figure 8:**
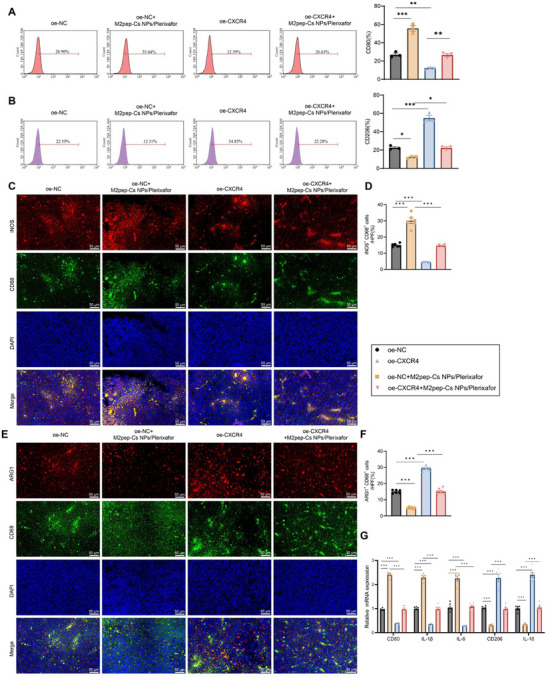
Effect of M2pep‐Cs NPs/Plerixafor nanoparticles on the reprogramming of TAMs via inhibition of the CXCL12–CXCR4 signaling pathway. Note: A) Flow cytometry analysis of CD80 expression in tumor tissues from each group of mice; B) flow cytometry analysis of CD206 expression in tumor tissues from each group of mice; C,D) immunofluorescence staining of iNOS protein expression in tumor tissues from each group of mice, scale bar: 50 µm; E,F) immunofluorescence staining of ARG1 protein expression in tumor tissues from each group of mice, scale bar: 50 µm; G) RT‐qPCR analysis of IL‐1β, IL‐6, TNFα, CD206, and IL‐10 mRNA levels in tumor tissues from each group of mice. Each group consisted of six mice, and values are presented as mean ± standard deviation. Multiple group comparisons were conducted using one‐way ANOVA, *p < 0.05, **p < 0.01, ***p < 0.001.

The M2pep‐Cs NPs/Plerixafor NPs inhibit the CXCL12–CXCR4 signaling pathway's impact on the reprogramming of TAMs. Flow cytometry results demonstrated that compared to the oe‐NC group, the oe‐NC+M2pep‐Cs NPs/Plerixafor group showed a significant increase in the M1 TAM marker CD80 in mouse tumor tissues, whereas the oe‐CXCR4 group showed a marked decrease. The oe‐CXCR4+M2pep‐Cs NPs/Plerixafor group exhibited a notable reduction in CD80 levels compared to the oe‐NC+M2pep‐Cs NPs/Plerixafor group (**Figure** **8**A). Compared to the oe‐NC group, the M2 TAMs cellular marker CD206 expression was significantly downregulated in the tumor tissues of mice in the oe‐NC+M2pep‐Cs NPs/Plerixafor group. In contrast, the proportion of CD206 was significantly upregulated in the tumor tissues of mice in the oe‐CXCR4 group; compared to the oe‐NC+M2pep‐Cs NPs/Plerixafor group, the proportion of CD206 was significantly upregulated in the tumor tissues of mice in the oe‐CXCR4+M2pep‐Cs NPs/Plerixafor group (Figure 8B). Immunofluorescence findings supported these observations: compared to the oe‐NC group, iNOS protein expression was significantly elevated in the oe‐NC+M2pep‐Cs NPs/Plerixafor group and reduced in the oe‐CXCR4 group. In addition, iNOS expression was lower in the oe‐CXCR4+M2pep‐Cs NPs/Plerixafor group compared to the oe‐NC+M2pep‐Cs NPs/Plerixafor group (Figure 8C,D). Similarly, ARG1 protein expression was significantly decreased in the oe‐NC+M2pep‐Cs NPs/Plerixafor group and increased in the oe‐CXCR4 group compared to the oe‐NC group; it was further elevated in the oe‐CXCR4+M2pep‐Cs NPs/Plerixafor group compared to the oe‐NC+M2pep‐Cs NPs/Plerixafor group (Figure 8E,F). RT‐qPCR results indicated that compared to the oe‐NC group, the mRNA levels of the M1 TAMs cytokines CD80, IL‐1β, and IL‐6 were significantly upregulated in the tumor tissues of mice in the oe‐NC+M2pep‐Cs NPs/Plerixafor group; while, the mRNA levels of the M2 TAMs cytokines CD206 and IL‐10 were significantly downregulated. In the oe‐CXCR4 group, CD80, IL‐1β, and IL‐6 mRNA levels were significantly downregulated; while, CD206 and IL‐10 were significantly upregulated. Compared to the oe‐NC+M2pep‐Cs NPs/Plerixafor group, the mRNA levels of CD80, IL‐1β, and IL‐6 were significantly downregulated, and the mRNA levels of CD206 and IL‐10 were significantly upregulated in the tumor tissues of mice in the oe‐CXCR4+M2pep‐Cs NPs/Plerixafor group (Figure 8G).

Finally, the M2pep‐Cs NPs/Plerixafor NPs inhibit the effects of the CXCL12–CXCR4 signaling pathway on the immune system. Flow cytometry results revealed that compared to the oe‐NC group, the number of T cells and B cells was significantly increased in the tumor tissues of mice in the oe‐NC+M2pep‐Cs NPs/Plerixafor group; while, in the oe‐CXCR4 group, the number of T cells and B cells was significantly decreased. Compared to the oe‐NC+M2pep‐Cs NPs/Plerixafor group, the number of T cells and B cells was significantly decreased in the tumor tissues of mice in the oe‐CXCR4+M2pep‐Cs NPs/Plerixafor group (Figure S13A, Supporting Information). Compared to the oe‐NC group, the number of CD4+ T cells and CD8+ T cells was significantly increased in the tumor tissues of mice in the oe‐NC+M2pep‐Cs NPs/Plerixafor group; while, these numbers were significantly decreased in the tumor tissues of mice in the oe‐CXCR4 group. Further, compared to the oe‐NC+M2pep‐Cs NPs/Plerixafor group, the number of CD4^+^ T cells and CD8^+^ T cells was significantly decreased in the tumor tissues of mice in the oe‐CXCR4+M2pep‐Cs NPs/Plerixafor group (Figure S13B, Supporting Information). Further analysis revealed that the number of CD4^+^ T cells outnumbered CD8^+^ T cells. Studies have shown that Th1 cells predominantly produce pro‐inflammatory cytokines such as IFN‐γ and IL‐12, which are involved in inflammatory and cellular immune responses, whereas Th2 cells produce anti‐inflammatory cytokines such as IL‐4, which are involved in humoral immune responses.^[^ ^37^ ^]^

Consequently, we further investigated the changes in the numbers of Th1 and Th2 cells within the CD4^+^ T cells. The results showed that compared to the oe‐NC group, the number of Th1 cells significantly increased; while, the number of Th2 cells significantly decreased in the tumor tissues of mice in the oe‐NC+M2pep‐Cs NPs/Plerixafor group. In contrast, in the tumor tissues of mice in the oe‐CXCR4 group, the number of Th1 cells significantly decreased, whereas the number of Th2 cells significantly increased. Further, compared to the oe‐NC+M2pep‐Cs NPs/Plerixafor group, the number of Th1 cells significantly decreased; while, the number of Th2 cells significantly increased in the tumor tissues of mice in the oe‐CXCR4+M2pep‐Cs NPs/Plerixafor group (Figure S13C,D, Supporting Information).

In addition, Western blot results indicated that compared to the oe‐NC group, the expression of pro‐inflammatory cytokines IFN‐γ, IL‐12, and IL‐6 increased in the tumor tissues of mice in the oe‐NC+M2pep‐Cs NPs/Plerixafor group. In contrast, the expression of IFN‐γ, IL‐12, and IL‐6 was significantly reduced in the tumor tissues of mice in the oe‐CXCR4 group. Further, compared to the oe‐NC+M2pep‐Cs NPs/Plerixafor group, the expression of IFN‐γ, IL‐12, and IL‐6 was significantly reduced in the tumor tissues of mice in the oe‐CXCR4+M2pep‐Cs NPs/Plerixafor group (Figure S13E, Supporting Information).

The results demonstrate that M2pep‐Cs NPs/Plerixafor NPs effectively modulate the CXCL12–CXCR4 signaling pathway, promoting the polarization of TAMs toward the pro‐inflammatory M1 phenotype; while, inhibiting the immunosuppressive M2 phenotype. This TAM reprogramming significantly enhances the immune system's capacity to recognize and eliminate GC cells. These findings underscore the therapeutic potential of M2pep‐Cs NPs/Plerixafor in anticancer immunomodulation, offering novel mechanistic insights and a promising nanotechnology‐based strategy for GC immunotherapy.

## 3 Discussion

This study highlights the unique therapeutic potential of self‐assembled M2pep‐Cs NPs/Plerixafor NPs in inhibiting the CXCL12–CXCR4 signaling pathway and reprogramming TAMs, thereby enhancing the immune system's capacity to recognize and eliminate GC cells (Table of Contents). Unlike conventional approaches, this research introduces a novel self‐assembling nanotechnology platform that optimizes drug delivery by improving nanoparticle stability, biocompatibility, and controlled release performance. A key innovation of this study lies in its multimodal assessment strategy, which simultaneously integrates genomic, proteomic, and metabolomic data to comprehensively elucidate the biological effects of the NPs. This approach surpasses the traditional focus on single‐pathway mechanisms and provides a more holistic view of the TME remodeling process. To our knowledge, such a system‐level evaluation of M2pep‐Cs NPs/Plerixafor has not been reported previously.

The CXCL12–CXCR4 signaling axis plays a critical role in regulating immune cell trafficking, tumor immune evasion, and the establishment of an immunosuppressive TME.^[^ ^38^ ^]^ In the context of GC, activation of this pathway has been shown to promote tumor cell survival, migration, and proliferation; while, concurrently suppressing anti‐tumor immune responses.^[^ ^23^, ^39^ ^]^ By selectively targeting and inhibiting this pathway, the present study demonstrated significant enhancement of M1‐type TAM polarization, which is closely associated with the initiation of robust anti‐tumor immunity. While prior research has indicated the therapeutic potential of CXCL12–CXCR4 blockade in cancer, this study advances the field by achieving more efficient and targeted modulation using a nanotechnology‐based delivery system.

M*Φ* plays a crucial role in shaping the tumor immune microenvironment, with their functional polarization states critically influencing immunotherapeutic outcomes. Specifically, the shift from the tumor‐supportive M2 phenotype to the pro‐inflammatory, anti‐tumor M1 phenotype is essential for enhancing anti‐tumor immunity.^[^ ^40^ ^]^ In the present study, the application of M2pep‐Cs NPs/Plerixafor NPs successfully induces this phenotypic reprogramming, representing a promising strategy for targeted macrophage modulation within the TME. Compared with existing nanomedicine approaches, this study introduces a novel self‐assembled nanoparticle platform with enhanced specificity, stability, and immune‐regulatory potential.

A major strength of this work lies in its multimodal analytical approach, which incorporates high‐throughput transcriptomics, proteomics, and metabolomics to comprehensively assess the biological effects of the NPs. This integrative strategy enables detailed mapping of the CXCL12–CXCR4 signaling pathway and downstream immune‐modulatory events, providing a system‐level understanding of how TAMs are reprogrammed. Such depth of analysis is rare in prior nanoparticle‐based immunotherapy research and offers valuable insight into the complexity of tumor‐immune dynamics.

While Plerixafor is clinically approved for hematopoietic stem cell mobilization by antagonizing CXCR4, our study specifically repurposes it in combination with M2pep‐Cs NPs to target CXCL12–CXCR4 signaling in TAMs. This dual‐function strategy not only disrupts tumor‐promoting chemokine signaling but also enhances immune recognition of GC cells. We acknowledge; however, that Plerixafor's systemic effects—particularly its influence on hematopoietic cell trafficking—may have contributed to some of the observed outcomes.

The therapeutic potential of M2pep‐Cs NPs/Plerixafor NPs demonstrated in this study provides compelling preclinical evidence for the development of next‐generation immunotherapies. By modulating M*Φ*function and remodeling the TME, this approach has the capacity to augment existing treatments, particularly in cases of recurrent or treatment‐resistant GC. Due to limitations in time and funding, we were unable to fully delineate whether the effects on TAM reprogramming resulted from direct versus indirect modulation of the CXCL12–CXCR4 axis. This remains an important question for future research. Moreover, translational validation in clinical settings—across various GC subtypes and disease stages—will be essential to confirm the safety, efficacy, and applicability of this therapeutic strategy.

Despite the significant insights and innovations presented in this study, several limitations should be acknowledged. First, only male mice were utilized to minimize potential variability introduced by hormonal cycles, which are known to influence both immune function and tumor progression. However, we recognize the importance of including both sexes in preclinical studies to uncover possible sex‐specific differences in therapeutic responses. Future investigations will incorporate female mice to assess whether the efficacy of M2pep‐Cs NPs/Plerixafor NPs varies by sex. While our self‐assembled NPs demonstrated encouraging in vitro and preliminary in vivo therapeutic activity, their long‐term safety, pharmacokinetics, and immunogenicity in humans remain to be fully elucidated. This study primarily focused on evaluating the NPs' ability to reprogram TAMs and suppress primary tumor growth via inhibition of the CXCL12–CXCR4 signaling pathway. This initial focus aimed to establish a mechanistic link between macrophage modulation and tumor suppression in vivo. Due to time and funding constraints, we did not assess the impact of M2pep‐Cs NPs/Plerixafor on tumor metastasis in this study. Given the well‐documented role of the CXCL12–CXCR4 axis in cancer metastasis, evaluating metastatic progression is a critical next step. We have therefore planned follow‐up experiments to include in vivo metastasis assays, which will provide a more comprehensive understanding of the therapeutic potential of our nanoparticle system. In addition, the complex and relatively costly synthesis of M2pep‐Cs NPs/Plerixafor may present challenges for widespread clinical application. To address this, future efforts will focus on simplifying the nanoparticle formulation, improving manufacturing scalability, and reducing production costs. Further studies are also required to thoroughly evaluate long‐term toxicity, potential off‐target effects, and systemic immune responses. It is important to note that this study emphasizes fundamental mechanistic exploration. The clinical applicability of M2pep‐Cs NPs/Plerixafor requires extensive validation through preclinical efficacy and safety studies, followed by well‐designed clinical trials.

Moving forward, future research should explore a broader array of nanoparticle formulations and optimize design parameters to improve therapeutic efficacy and cost‐effectiveness. In addition, expanding the application of this nanotherapeutic strategy to other tumor types will help determine its universality and translational potential across cancer types. In conclusion, this study introduces a novel nanotechnology‐based approach for the treatment of GC, focusing on immune modulation through TAM reprogramming via the CXCL12–CXCR4 signaling axis. These findings provide a strong foundation for developing new classes of immuno‐nanotherapeutics, and ongoing optimization may significantly contribute to more effective and personalized cancer treatment strategies in the future.

## 4 Experimental Section

### Preparation of M2pep‐Cs NPs/Plerixafor NPs

The synthesis of M2pep‐Cs NPs/Plerixafor NPs began with the preparation of chitosan (Cs) NPs. Briefly, 3 mL of acetone (650501, Sigma–Aldrich) was gradually added to 2 mL of an aqueous Cs solution (1105508, Sigma–Aldrich, 5 mg mL^−1^) containing 1.25 mg of disodium EDTA (EDTA·2Na; 324503, Sigma–Aldrich). This process yielded a suspension of milky‐white, non‐crosslinked NPs. Crosslinking was then initiated by adding 20 µL of glutaraldehyde solution (8.20603, Sigma–Aldrich), followed by incubation for 4 h. The resulting Cs NPs were purified via dialysis against distilled water for 48 h using a dialysis membrane (molecular weight cutoff 14 kDa; FDM514m, Beyotime). The M2pep peptide (sequence: YEQDPWGVKWWY), labeled with Cy5, was conjugated to the surface of the Cs NPs. Specifically, 0.5 mg of M2pep was added to 1.0 mL of Cs NPs (1.0 mg mL^−1^) and stirred at 200 rpm at room temperature for 16 h. The NPs were then centrifuged at 10 000 rpm for 15 min, washed three times with deionized water, and resuspended after each wash. The conjugation efficiency, determined using UV–visible spectrophotometry at 280 nm, was ≈87%.^[^ ^41^ ^]^

Plerixafor loading was performed by adding 200 µL of a 2 mg Plerixafor solution (HY‐10046, MedChemExpress) to 1 mL of the M2pep‐Cs NPs suspension (10 mg). The mixture was stirred at room temperature in the dark for 12 h. Following incubation, unbound Plerixafor was removed by centrifugation at 10 000 rpm for 10 min. The pellet was resuspended in 2 mL of deionized water to yield the final Plerixafor‐loaded formulation, termed M2pep‐Cs NPs/Plerixafor. The concentration of unbound Plerixafor in the supernatant was quantified at 425 nm using a UV spectrophotometer (UV‐1900, Shimadzu). The drug loading content (DLC) and drug loading efficiency (DLE) were calculated using the following formulas: DLC (%) = (amount of drug loaded)/(total weight of nanoparticles) × 100%; DLE (%) = (amount of drug loaded)/(total amount of drug) × 100%.

The hydrodynamic size, polydispersity index (PDI), and zeta potential of the NPs were measured via dynamic light scattering (DLS) using a Nano‐ZS90 particle size and zeta potential analyzer (Malvern Instruments, UK) equipped with a 633 nm He–Ne laser. All measurements were conducted at 25 °C and repeated in triplicate. To evaluate physiological stability, NPs were resuspended in various media—including deionized water, saline, phosphate‐buffered saline (PBS), and RPMI‐1640 medium (11875101, Gibco, USA)—and incubated for 24 h. In addition, M2pep‐Cs NPs were incubated in solutions of different pH values (4.5, 5.5, 6.5, 7.5, and 8.5) for 24 h to assess pH‐dependent stability. Post‐incubation, nanoparticle size, distribution, and scattering intensity were analyzed using DLS. Nanoparticle morphology was visualized using transmission electron microscopy (TEM) with a Hitachi H7650 instrument (Hitachi, Japan). Detailed methodological procedures are illustrated in Figure S1, Supporting Information.

### In Vitro Release of Plerixafor

The release profile of Plerixafor from M2pep‐Cs NPs was evaluated under three different pH conditions: 7.4, 6.5, and 5.0. For the release study, 1 mL of Plerixafor‐loaded NPs was placed into a dialysis bag (molecular weight cutoff 3.5 kDa) and immersed in 5 mL of PBS. Each dialysis bag was then transferred to a 50 mL centrifuge tube and maintained at 37 °C under gentle stirring (100 rpm) in the dark. At predetermined time intervals, 5 mL of the external PBS was collected and replaced with an equal volume of fresh PBS to maintain sink conditions. The concentration of Plerixafor released into the buffer was quantified using UV spectrophotometry at 425 nm.

### Cellular Uptake

To assess nanoparticle uptake, RAW264.7 cells were seeded in 12‐well plates and treated with 10 µm of free Plerixafor, M2pep‐Cs NPs (10 µm), or M2pep‐Cs NPs/Plerixafor (10 µm) for 4 h. After incubation, the cells were washed three times with PBS and fixed with cold paraformaldehyde for 15 min. Following fixation, the nuclei were stained with DAPI for 10 min. Cellular internalization was visualized using a confocal laser scanning microscope (Leica, CH), and fluorescence intensity was quantified using ImageJ software. In addition, the treated cells were subjected to flow cytometry analysis. After fixation and DAPI staining, samples were analyzed using a flow cytometer (FC500, Beckman Coulter, USA). All experiments were performed in triplicate.

### In Vitro Biocompatibility Assessment: Live/Dead Cell Staining and CCK‐8 Assay

RAW264.7 macrophages were seeded at a density of 1 × 10^6^ cells per well in 12‐well plates and co‐cultured with Cs NPs, M2pep‐Cs NPs, or M2pep‐Cs NPs/Plerixafor (all at 10 µm) for 1, 2, and 3 days. A Live/Dead staining solution was prepared by mixing PBS, calcein‐AM, and propidium iodide (PI) at a ratio of 1 mL:3 µL:5 µL (ZY140632, Zeye Biotechnology, China) and applied to each well. After 37 min of incubation at 30 °C, the samples were imaged using a fluorescence microscope (IMT‐2, Olympus, Japan). Live cells exhibited green fluorescence; while, dead cells were stained red. Quantification was performed by counting cells in randomly selected fields of view.

For cytotoxicity evaluation, RAW264.7 cells (1 × 10^6^ per well) were co‐incubated with Cs NPs, M2pep‐Cs NPs, or M2pep‐Cs NPs/Plerixafor (10 µm) for 1, 2, and 3 days. At each time point, 100 µL mL^−1^ of CCK‐8 solution (C0037, Beyotime) was added to each well and incubated for 2 h. Subsequently, 100 µL of supernatant was transferred to a 96‐well plate, and absorbance was measured at 450 nm using a microplate reader (BioTek Synergy Neo2 Hybrid, Agilent, USA).

### Hemolysis Assay

To evaluate the hemocompatibility of M2pep‐Cs NPs/Plerixafor NPs, a hemolysis assay was performed at varying nanoparticle concentrations ranging from 100 to 3.13 µg mL^−1^. PBS served as the negative control; while, deionized water was used as the positive control. Fresh human whole blood containing EDTA as an anticoagulant (Innovative Reagents for Innovative Research, Suzhou, China) was mixed with each nanoparticle concentration and control solution. Samples were incubated at 37 °C for 3 h, followed by centrifugation at 2000 × *g* for 10 min to separate cellular debris from plasma. The supernatant's absorbance at 540 nm was measured using a spectrophotometer to quantify released hemoglobin and assess hemolysis.

### In Vitro Cell Culture

The following cell lines were used: murine macrophages (RAW264.7; TIB‐71, ATCC, USA), human embryonic kidney cells (HEK‐293T; CRL‐3216, ATCC), and mouse GC cells (MFC; JSY‐CC3729, Jscall, China). Cells were cultured in DMEM/F‐12 medium supplemented with 10% FBS. All cell culture reagents, including FBS (10099), RPMI‐1640, and DMEM/F‐12 (21041025), were purchased from Gibco (USA).

To induce macrophage (M*Φ*) differentiation, RAW264.7 cells were treated with 320 nm phorbol 12‐myristate 13‐acetate (PMA; 16561‐29‐8, Sigma–Aldrich) for 24 h. For M2‐type macrophage (M2 M*Φ*) polarization, THP‐1 cells were first treated with 320 nm PMA for 6 h; then, cultured with IL‐4 (20 ng mL^−1^; 200–04, PeproTech) and IL‐13 (20 ng mL^−1^; 200–13, PeproTech) for 18 h. Cellular morphology and M2 surface marker expression were subsequently confirmed.^[^ ^42^ ^]^

To evaluate interactions between macrophages and tumor cells, a Transwell co‐culture system was employed. MFC cells (1 × 10^5^) were seeded in the upper chamber of Falcon Cell Culture Inserts (Corning, NY, USA); while, pretreated RAW264.7 macrophages (1 × 10^6^) were seeded in the lower chamber. For invasion assays, RAW264.7 cells were placed in the lower chamber containing RPMI with 10% FBS or serum‐free medium, and serum‐free MFC cells (2 × 10^5^) were seeded in the upper chamber. Cell invasion was assessed after 24 h.^[^ ^43^ ^]^

The CXCL12 overexpression plasmid was constructed using the pCMV6‐AC‐GFP vector (LM‐2069, LMAI Bio, Shanghai, China), synthesized by Sangon Biotech (Shanghai, China). CXCR4 overexpression (oe‐CXCR4) and control (oe‐NC) lentiviruses were generated using HEK293T cells (Bio‐72947, biobw). Plasmids containing the luciferase reporter gene (oe‐NC, oe‐CXCR4) and helper plasmids were co‐transfected into HEK293T cells using Lipofectamine 2000 (11668030, Thermo Fisher, USA). Lentiviral packaging, expansion, and purification were performed by Sangon Biotech. For transduction, RAW264.7 cells (5 × 10^5^) were seeded in 6‐well plates. When the cells reached 70–90% confluence, they were infected with the packaged lentivirus at a multiplicity of infection (MOI) of 10 (titer ≈ 5 × 10^6^ TU mL^−1^), supplemented with 5 µg mL^−1^ polybrene (TR‐1003, Sigma–Aldrich, UK). After 4 h, an equal volume of medium was added to dilute polybrene. The medium was replaced after 24 h, and luciferase expression was observed at 48 h to assess transfection efficiency. Stable cell lines were selected using puromycin (E607054, Sangon Biotech, Shanghai, China). Cells were harvested upon survival in a puromycin‐containing medium, and overexpression efficiency was confirmed via RT‐qPCR.^[^ ^44^ ^]^ The procedure is illustrated in Figure S2, Supporting Information.

Macrophage experimental groups included: M*Φ* group: M*Φ*, M2 group: M2‐type M*Φ*, Plerixafor group: M*Φ* or M2‐type M*Φ* treated with 10 µm Plerixafor for 24 h, M2pep‐Cs NPs group: M*Φ* or M2‐type M*Φ* treated with 10 µm M2pep‐Cs NPs for 24 h, and M2pep‐Cs NPs/Plerixafor group: M*Φ* or M2‐type M*Φ* treated with 10 µm M2pep‐Cs NPs/Plerixafor for 24 h.

The co‐culture groups of M*Φ* with MFC cells were as follows: oe‐NC group: cells co‐cultured with overexpression control lentivirus, oe‐NC + M2pep‐Cs NPs/Plerixafor group: cells co‐cultured with overexpression control lentivirus and treated with 10 µm M2pep‐Cs NPs/Plerixafor, oe‐CXCR4 group: cells co‐cultured with overexpression CXCR4 lentivirus, and oe‐CXCR4 + M2pep‐Cs NPs/Plerixafor group: cells co‐cultured with overexpression CXCR4 lentivirus and treated with 10 µm M2pep‐Cs NPs/Plerixafor.

### Immunofluorescence Staining

Immunofluorescence staining was performed to evaluate protein expression in RAW264.7 macrophages, MFC GC cells, and tumor tissue sections. Samples were fixed with 4% paraformaldehyde at room temperature for 15 min, followed by two washes with PBS. Permeabilization was carried out using 0.5% Triton X‐100 (P0096, Beyotime Biotechnology, China) for 10 min. To block nonspecific binding, samples were incubated in PBS containing 5% bovine serum albumin (BSA) for 30 min. After blocking, cells and tissues were incubated overnight at 4 °C with the following primary antibodies: CD86 (MA1‐10299, 1:200), CD206 (MA5‐16871, 1:200), CXCL12 (PA5‐114344, 1:100), CXCR4 (704015, 1:500), iNOS (MA5‐17139, 5 µg mL^−1^), ARG1 (702730, 1:100), and CD68 (14‐0681‐82, 5 µg mL^−1^). After three PBS washes, samples were stained with FITC‐conjugated phalloidin (F432, Invitrogen) or rhodamine‐conjugated phalloidin (R415, Invitrogen) to label the cytoskeleton. Subsequently, samples were incubated for 1 h at room temperature with secondary antibodies conjugated to Alexa Fluor 647 (ab150083, 1:200) or Alexa Fluor 488 (ab150077, 1:200). Following secondary antibody incubation, samples were washed three times with PBS and counterstained with DAPI (D3571, 10 µg mL^−1^) for 10 min at room temperature to visualize nuclei. Stained samples were stored at 4 °C until analysis. Imaging was performed using a fluorescence microscope (IMT‐2, Olympus, Japan). The fluorescence intensity of target proteins relative to nuclear staining was quantified using ImageJ software. For cellular assays, five sections were examined per sample across six to ten randomly selected fields. All experiments were independently repeated three times. Alexa Fluor 647 and Alexa Fluor 488 were obtained from Abcam (UK); all other antibodies were purchased from Invitrogen (USA).

### Flow Cytometry Analysis and Cell Apoptosis Assay

RAW264.7 cells and tumor tissues were digested with 0.25% trypsin (25200072, Gibco, USA) and counted. Approximately 1 × 10^6^ cells were resuspended in 200 µL of fluorescence‐activated cell sorting (FACS) buffer (660585, BD Biosciences, USA), followed by incubation with 2 µL of fluorescently labeled antibodies on ice for 30 min. To minimize non‐specific binding, cells were subsequently incubated in PBS containing 5% BSA for 30 min. After washing with FACS buffer, cells were fixed in 10% formalin (R04587, Merck, USA) and analyzed using a BD FACSCalibur flow cytometer (BD Biosciences, USA) to determine the percentage of antigen‐positive cells. The antibodies used included CD86 (12‐0862‐82, 0.125 µg per test, Invitrogen, USA), CD80 (ab307467, 1:500, Abcam, USA), and CD206 (17‐2061‐82, 0.25 µg per test, Invitrogen, USA).

Single‐cell suspensions from tumor tissues were prepared via enzymatic digestion using collagenase and DNase for 1–2 h, followed by density gradient centrifugation. The resulting cells were adjusted to ≈1 × 10^6^ cells mL^−1^ and stimulated with a cocktail containing PMA (HY‐18739, MedChemExpress, Shanghai, China), ionomycin (HY‐13434, MedChemExpress, Shanghai, China), and GolgiPlug (Brefeldin A; 555029, BD Biosciences, USA) for 5 h at 37 °C in a humidified atmosphere with 5% CO_2_. To block Fc receptor‐mediated non‐specific binding, cells were pre‐incubated with CD16/CD32 antibodies (0.5 mg mL^−1^; 14‐0161‐82, Thermo Fisher, USA) for 10 min prior to surface staining. The following antibodies were used for immune phenotyping: APC‐labeled CD19 antibodies for B cells (0.2 mg mL^−1^, 17‐0193‐82, Thermo Fisher, USA), PE‐labeled CD3 antibodies for T cells (0.2 mg mL^−1^, 12‐0031‐82, Thermo Fisher, USA), FITC‐labeled CD4 antibodies for CD4^+^ T cells (0.5 mg mL^−1^, 11‐0041‐82, Thermo Fisher, USA), PE‐Cy7 labeled CD8 antibodies for CD8^+^ T cells (0.2 mg mL^−1^, A15385, Thermo Fisher, USA), APC‐labeled anti‐IFN‐γ antibodies for Th1 cells (0.2 mg mL^−1^, 17‐7311‐82, Thermo Fisher, USA), and PE‐labeled anti‐IL‐4 antibodies for Th2 cells (0.2 mg mL^−1^, 12‐7041‐82, Thermo Fisher, USA). Flow cytometric analysis was performed using appropriate gating strategies to accurately distinguish immune cell subsets. Unstained and single‐stained controls were used to calibrate gating and ensure the specificity of the fluorescence signals. All procedures were carried out under sterile conditions to avoid contamination.

Apoptosis in MFC cells was assessed using Annexin V‐FITC/PI double staining. MFC cells (2 × 10^5^ per well) were seeded into 6‐well plates and treated according to the experimental protocol. Following treatment, cells were harvested using trypsin (R001100, Gibco, USA), collected in 15 mL centrifuge tubes, and centrifuged at 800 × *g* to remove the supernatant. Apoptosis was evaluated using a commercial detection kit (556547, BD Biosciences, USA) following the manufacturer's instructions. Briefly, the cell pellet was resuspended in 500 µL of binding buffer, followed by the addition of 5 µL of Annexin V‐FITC and 5 µL of PI. Samples were incubated in the dark for 15 min at room temperature; and then, analyzed using a BD FACSCalibur flow cytometer. Flow cytometry plots were interpreted as follows: the upper left quadrant represented necrotic cells, the upper right quadrant indicated late apoptotic cells, the lower right quadrant showed early apoptotic cells, and the lower left quadrant denoted viable cells. The total apoptosis rate was calculated as the sum of early and late apoptotic cell percentages (i.e., upper right + lower right quadrants). Each experiment was independently repeated three times.

### Enzyme‐Linked Immunosorbent Assay (ELISA)

Culture supernatants from RAW264.7 cells and homogenized tumor tissue lysates were collected and appropriately diluted to ensure that target protein concentrations fell within the linear range of the standard curves. The levels of CXCL12 and CXCR4 in both supernatants and tissue lysates were quantified using commercially available ELISA kits for CXCL12 (ab100741, Abcam) and CXCR4 (ab309218, Abcam). For CXCR4 detection, 96‐well ELISA plates were coated with a capture antibody solution specific to CXCR4 and incubated overnight at 4 °C. Following incubation, wells were washed thoroughly to remove unbound material. An enzyme‐conjugated secondary antibody was then added, and unbound antibodies were removed through repeated washing. A substrate solution was added to initiate a colorimetric reaction, which was allowed to proceed for a defined period before being terminated with a stop solution. The absorbance of each well was measured using a microplate reader, and the concentrations of CXCL12 and CXCR4 were calculated based on their respective standard curves.^[^ ^45^ ^]^

### Construction of GC Mouse Model

A total of 51 healthy male C57BL/6J mice (4–6 weeks old) were obtained from Charles River (China) and housed individually in a specific pathogen‐free (SPF) animal facility. Environmental conditions were maintained at 60–65% humidity and a temperature of 22–25 °C, with a 12‐h light/dark cycle. All animals had ad libitum access to sterilized food and water. Mice were acclimatized for 1 week prior to experimentation, during which their health status was carefully monitored. All procedures involving animals were approved by the Institutional Animal Care and Use Committee (IACUC) and conducted in accordance with the Guide for the Care and Use of Laboratory Animals (NIH, USA) and the ethical standards of the National Academy of Sciences (USA).

Prior to tumor implantation, MFC GC cells were transfected with a firefly luciferase reporter plasmid (D2102, Beyotime, Shanghai, China), and stable clones were selected for further use. RAW264.7 macrophages and MFC cells were mixed at a 1:1 ratio and suspended in a 1:1 v/v mixture of PBS containing 50% FBS and Matrigel. A total of 2 × 10^5^ cells of each type (RAW264.7 and MFC) were injected twice weekly into the gastric wall of 6‐week‐old female C57BL/6J mice to establish an orthotopic GC model.

Once tumor volumes reached ≈100 mm^3^, mice were randomly assigned to treatment groups. Mice in the treatment group received intravenous injections of 10 µm M2pep‐Cs NPs/Plerixafor twice per week for 4 consecutive weeks. At the end of the treatment period, animals were anesthetized using 2% isoflurane in oxygen (1 L min^−1^; R510‐22‐10, RWDLS, Shenzhen, China) following intraperitoneal injection of 150 mg kg^−1^ luciferin (ST196, Beyotime, Shanghai, China). After a 10‐min stabilization period, tumor growth was monitored using an IVIS Lumina Series in vivo imaging system (PerkinElmer, USA). Following imaging, mice were euthanized via CO_2_ inhalation. Tumor tissues were either fixed in formalin and paraffin‐embedded or snap‐frozen in liquid nitrogen and stored at −80 °C for subsequent analyses.^[^ ^46^ ^]^

The mice were randomly assigned to the following experimental groups: normal group: injection of 200 µL PBS. GC group: injection of 200 µL MFC cells. M2pep‐Cs NPs/Plerixafor group: Injection of 200 µL MFC cells and 100 µL of 10 µm M2pep‐Cs NPs/Plerixafor into GC mice. oe‐NC group: injection of 200 µL overexpression control lentivirus RAW264.7 cells and MFC cells followed by 100 µL PBS. oe‐NC + M2pep‐Cs NPs/Plerixafor group: injection of a mixture of 200 µL overexpression control lentivirus RAW264.7 cells and MFC cells followed by 100 µL of 10 µm M2pep‐Cs NPs/Plerixafor into GC mice. oe‐CXCR4 group: injection of 200 µL overexpression CXCR4 lentivirus RAW264.7 cells and MFC cells followed by 100 µL PBS. oe‐CXCR4 + M2pep‐Cs NPs/Plerixafor group: injection of a mixture of 200 µL overexpression CXCR4 lentivirus RAW264.7 cells and MFC cells followed by 100 µL of 10 µm M2pep‐Cs NPs/Plerixafor into GC mice. A schematic of the animal experiment design is provided in Figure S3, Supporting Information.

### Hematoxylin and Eosin (H&E) Staining

Mouse tumor tissues fixed in paraformaldehyde were dehydrated, embedded in paraffin, and sectioned at a thickness of 5 µm. Sections were collected from both internal and external tumor regions at 200 µm intervals. For histological analysis, selected slides were deparaffinized in xylene and rehydrated through a graded ethanol series followed by distilled water. H&E staining was performed by immersing the sections in Harris hematoxylin (ST2067, Beyotime) for 5 min. Sections were then differentiated in 0.5% hydrochloric acid in ethanol (C0165S, Beyotime) for 10 s and subsequently stained with eosin solution (C0109, Beyotime) for 40 s. After dehydration and clearing, the slides were mounted using neutral resin and examined under a light microscope.

### TUNEL Staining

Apoptotic cells in mouse tumor tissues were identified using a TUNEL apoptosis detection kit (C1086, Beyotime, China). Tissue sections were incubated with 50 µL of TUNEL reagent in the dark at 37 °C for 60 min. Nuclei were counterstained with DAPI (1 µg mL^−1^) for 30 min, followed by three washes with PBS. Imaging was performed at 400× magnification using a fluorescence microscope equipped with a digital camera (BX53, Olympus, Japan). The ratio of TUNEL‐positive fluorescence to total DAPI‐stained nuclei was calculated using ImageJ software. Each experimental group consisted of five mice, with five sections prepared per mouse. Representative images were captured for quantitative analysis.

### Immunohistochemistry (IHC)

Tumor tissue samples were fixed in 4% paraformaldehyde, dehydrated, cleared, embedded in paraffin, and sectioned at a standard thickness. For IHC analysis, tissue sections were deparaffinized, rehydrated, and subjected to antigen retrieval using 1% hydrogen peroxide. Slides were placed in PBS and heated in a microwave until boiling to unmask antigenic sites. Staining was conducted using a universal two‐step detection kit (PV‐9000, ProteinTech, USA) following the manufacturer's instructions. The primary antibody used was anti‐Ki67 (ab15580, 1:500; Abcam). Visualization was performed under a light microscope (CX43, Olympus, Japan). Positive immunoreactivity appeared as light brown to brownish‐yellow staining. Each experimental group included six mice, with five sections analyzed per mouse. Six random fields of view were selected per section. Quantitative analysis of IHC was performed using ImageJ software.

### Biodistribution of the Nanovaccine In Vivo

To evaluate the in vivo biodistribution of M2pep‐Cs NPs/Plerixafor, Cy5‐labeled M2pep peptide‐modified NPs were administered to GC mice via tail vein injection once tumor volumes reached ≈100 mm^3^. Whole‐body fluorescence imaging was performed at predefined time points using the IVIS imaging system (PerkinElmer, USA) to monitor the real‐time distribution of the NPs. After 7 days, tumor tissues and major organs were harvested for ex vivo fluorescence imaging. In addition, confocal laser scanning microscopy (CLSM) was used to further investigate the tissue‐level localization and distribution of M2pep‐Cs NPs/Plerixafor within tumor and organ samples.

### High‐Throughput Transcriptome Sequencing

For transcriptomic profiling, tumor tissues were collected from both the GC group (*n* = 3) and the M2pep‐Cs NPs/Plerixafor treatment group (*n* = 3). Library preparation and RNA sequencing were performed by CapitalBio Technology (Beijing, China). Total RNA (5 µg per sample) was extracted and processed for sequencing. Ribosomal RNA (rRNA) was depleted using the Ribo‐Zero Magnetic Kit (MRZG12324, Epicentre, USA). Sequencing libraries were constructed using the NEBNext Ultra RNA Library Prep Kit for Illumina (E7760S, NEB, USA). Briefly, RNA was fragmented to ≈300 bp in NEB Next First Strand Synthesis Reaction Buffer (5×). First‐strand cDNA was synthesized using random hexamer primers, followed by second‐strand cDNA synthesis with a dUTP‐containing buffer to maintain strand specificity. End repair, poly(A) tailing, and adapter ligation were subsequently performed. USER Enzyme (M5508, NEB, USA) was used to digest the second cDNA strand. The libraries were then amplified and purified via PCR. Library quality and fragment size distribution were assessed using an Agilent 2100 Bioanalyzer. Quantification was performed using the KAPA Library Quantification Kit (kk3605, Merck, USA). Paired‐end sequencing was carried out on the Illumina NextSeq 500 platform.

### Transcriptome Sequencing Data Analysis

Quality control of raw paired‐end sequencing reads was performed using FastQC (v0.11.8). Sequencing adapters and poly(A) tails were removed using Cutadapt (v1.18). Reads containing more than 5% ambiguous nucleotides (N) were filtered out using custom Perl scripts. High‐quality reads, defined as those with ≥ 70% of bases having a Phred score > 20, were retained using FASTX‐Toolkit (v0.0.13). Paired‐end read repair was conducted using BBMap tools. Filtered reads were aligned to the mouse reference genome using HISAT2 (v0.7.12).

Differential expression analysis was conducted using the edgeR package in R. Genes were considered differentially expressed if they met the criteria of |log_2_FC| > 1 and *p* < 0.05. Heatmaps of intersecting differentially expressed genes were generated using the heatmap package in R.

Functional enrichment analysis was conducted using Gene Ontology (GO) and the Kyoto Encyclopedia of Genes and Genomes (KEGG) pathway annotations via the “clusterProfiler,” “org.Hs.eg.db,” “enrichplot,” “DOSE,” and “ggplot2” packages in R.

### Integrated Database Analysis of the CXCL12/CXCR4 Signaling Pathway in GC

To investigate the immunological relevance of the CXCL12–CXCR4 signaling axis in GC, its association with immune cell infiltration—particularly with CD8^+^ T cells and macrophages—was analyzed using the TIMER database (https://cistrome.shinyapps.io/timer/). Additional correlation analyses between CXCL12/CXCR4 signaling and various T cell subtypes were performed using the TISIDB platform (http://cis.hku.hk/TISIDB/).

### Quantitative Proteomics Analysis

Tumor tissues from the GC group (*n* = 3) and the M2pep‐Cs NPs/Plerixafor group (*n* = 3) were subjected to quantitative proteomic analysis. Tissue lysates were prepared using RIPA buffer containing protease inhibitors. Sonication was performed three times at 30‐s intervals every 5 min to ensure complete cell lysis. Protein concentrations were measured using the BCA Protein Assay Kit (23225, Thermo Fisher, USA) and adjusted to fall within the standard curve range.

Following pH adjustment to 8.0, proteins were digested with trypsin at a 1:50 enzyme‐to‐protein ratio and incubated at 37 °C for 16 h. The resulting peptides were purified using ZipTip C18 columns and labeled using iTRAQ reagents. Desalted peptides were analyzed by LC‐MS/MS using a QSTAR Elite Hybrid mass spectrometer (Applied Biosystems/MDS‐SCIEX) coupled to an online HPLC system (Shimadzu, Japan).

For each sample, 30 µL of peptide solution was injected and separated on a nano‐bore C18 column (75 µm ID × 15 cm, 5 µm particle size; New Objectives, Woburn, MA). A 90‐min linear gradient was employed using mobile phase A (0.1% formic acid [FA]/2% acetonitrile [ACN]) and mobile phase B (0.1% FA/100% ACN) at a flow rate of 0.2 µL min^−1^. The electrospray interface operated at a constant 30 µL min^−1^. The instrument operated in positive ion mode with a scan range of 300–2000 m z^−1^, selecting precursor ions with charge states from +2 to +4 for fragmentation. The three most intense precursor ions exceeding a threshold of five counts were selected for MS/MS. Dynamic exclusion was applied for 30 s with a mass tolerance of 30 mDa.

Data acquisition was performed using automated collision energy and Smart IDA (information‐dependent acquisition) protocols. The fragment intensity multiplier was set to 20, and the maximum accumulation time was 2 s. Each sample was analyzed in triplicate to ensure reproducibility (technical replicates = 3). Validation of proteomics results was conducted via Western blotting using the same tissue samples. Parameters were set as follows: 1) MS: scan range (m/z) = 350–1500, resolution = 1 20 000, automatic gain control (AGC) target = 4e^5^, and maximum injection time = 50 ms; 2) HCD‐MS/MS: resolution = 30 000, AGC target = 1e^5^, and collision energy = 33; and 3) data‐independent acquisition (DIA): each window overlapped by 1m/z with a total of 47 windows. The iRT calibration kit (Ki3002, Biognosys AG, Switzerland) was added to calibrate the retention times of extracted peptides. DIA datasets were processed using Spectronaut V13 (Biognosys AG, Switzerland), including normalization and relative quantification. Differentially expressed proteins were identified using Welch's ANOVA, with statistical thresholds set at *p* < 0.05 and |log_2_FC| > 1.0.

### Metabolomic Analysis

Tumor tissues from the GC group (*n* = 6) and from the M2pep‐Cs NPs/Plerixafor treatment group (*n* = 6) were collected for metabolomic profiling. A 300 µL aliquot of each tissue sample was transferred to a 1.5 mL polypropylene tube, mixed with 900 µL of 80% methanol containing 0.1% formic acid (FA), and vortexed for 2 min. The mixtures were then centrifuged at 12 000 × *g* for 10 minutes at 4 °C. Supernatants were collected and transferred into autosampler vials for subsequent analysis.

Metabolomic analysis was conducted using a Shimadzu LC‐20 UHPLC system coupled with an AB Sciex Triple TOF‐6600 mass spectrometer. Chromatographic separation was achieved on a Waters ACQUITY UPLC HSS T3 C18 column (100 × 2.1 mm, 1.8 µm) maintained at 40 °C, with a flow rate of 0.4 mL min^−1^. The mobile phase consisted of ACN and water containing 0.1% FA. The gradient elution program for mobile phase B was as follows: 5% (0.0–11.0 min), linear increase to 90% (11.0–12.0 min), and return to 5% (12.1–14.0 min). The eluate was directly introduced into the mass spectrometer without flow splitting.

Mass spectrometric parameters were as follows: ionization voltage at 5500 V, capillary temperature at 550°C, spray gas flow at 50 psi, and auxiliary heating gas flow at 60 psi. The data were preprocessed and subjected to Orthogonal Partial Least Squares‐Discriminant Analysis (OPLS‐DA) with 100 permutation tests to prevent model overfitting. Metabolites with a Variable Importance in Projection (VIP) score >1 and *p* < 0.05 in the OPLS‐DA model were identified as differential metabolites (DMs). Further univariate analysis using Student's t‐test was performed, and metabolites with fold changes ≥1 or ≤0.5 and *p* < 0.05 were designated as final DMs. Relevant metabolic pathways were identified using MetaboAnalyst (v5.0). The complete workflow is illustrated in Figure S4.

### Immunofluorescence Detection of EdU‐Positive Cells

Cell proliferation was evaluated using the EdU‐488 Cell Proliferation Assay Kit (C0071S, Beyotime, China) according to the manufacturer's protocol. After EdU incorporation, cell nuclei were stained with Hoechst 33342. Fluorescence images were acquired using a fluorescence microscope (IMT‐2, Olympus, Japan). Proliferation was quantified by calculating the ratio of EdU‐positive cells to total Hoechst‐stained nuclei using ImageJ software.

### Scratch Test

MFC cells were seeded into 6‐well plates and grown to full confluence. A sterile 200 µL pipette tip was used to create a straight scratch in the monolayer. Detached cells were removed by rinsing three times with PBS. To prevent cell proliferation, cells were pretreated with mitomycin C (1 µg mL^−1^; M5353, Sigma–Aldrich) for 1 h prior to scratching. Wound closure was monitored and photographed at 0, 24, and 48 h. The initial scratch width at time 0 (*T* = 0 h) and residual widths at 24 h (*T* = 24 h) and 48 h (*T* = 48 h) were measured using image analysis software. Cell migration was quantified by calculating the percentage reduction in scratch width at each time point. For each experimental condition, five independent wells were analyzed, with six random fields of view per well. The experiment was conducted in triplicate.

### Transwell Assay

Transwell assays were performed using polycarbonate membrane cell culture inserts (CLS3422, Corning, USA) and BioCoat Matrigel invasion chambers with 8.0 µm PET membranes (354480, Corning, USA). For invasion assays, MA782/5s‐8101‐R cells (1 × 10^4^) were seeded into the upper chambers pre‐coated with 50 µL of Matrigel (354234, BD Biosciences, USA), which was allowed to solidify at 37 °C for 30 min. The lower chambers were filled with DMEM supplemented with 10% FBS to serve as a chemoattractant. For migration assays, MFC cells (1 × 10^4^) were seeded in uncoated inserts; while, RAW264.7 macrophages were placed in the lower chambers. After 16–24 hours of incubation, non‐migrated cells on the upper surface of the membrane were gently removed with a sterile cotton swab. Cells that had migrated or invaded through the membrane were fixed with 4% paraformaldehyde, stained with crystal violet (C0121, Beyotime, China), and visualized under an inverted microscope (XDS‐900, Caikon, China). For each cell type, five replicates were prepared, with six random fields of view analyzed per insert. The number of migrated or invaded cells was quantified using the “Analyze Particles” function in ImageJ. All experiments were independently repeated three times.

### Western Blot Analysis

Protein samples from cells and tumor tissues were extracted using RIPA lysis buffer (P0013B, Beyotime Biotechnology, China). Protein concentrations were determined using the BCA Protein Assay Kit (A53226, Thermo Fisher Scientific, USA). Equal amounts of protein were separated by SDS‐PAGE and transferred onto PVDF membranes (PVH85R, Millipore, Germany) using a wet transfer system. Membranes were blocked in 10% BSA (37520, Thermo Fisher Scientific, USA) for 1 h at room temperature and incubated overnight at 4 °C with primary antibodies: CXCL12 (PA5‐114344, 1:500), CXCR4 (704015, 1:500), IFN‐γ (AMC4739, 1:1000), IL‐12 (16‐7123‐81, 1:1000), IL‐6 (M620, 1:1000), and GAPDH (MA5‐15738, 1:1000). After washing, membranes were incubated with HRP‐conjugated secondary antibodies (AN‐170‐250UG, 1:5000) for 2 h. Bands were visualized using a Syngene G:BOX F3 imaging system (Antpedia, China), and band intensity was quantified using ImageJ software. The grayscale values of target proteins were normalized to GAPDH. All cell experiments were performed in triplicate, and each animal experiment included six mice per group. All antibodies were obtained from Invitrogen.

### RT‐qPCR Analysis

Total RNA from cells and tumor tissues was extracted using TRIzol reagent (15596026, Thermo Fisher, USA). RNA concentration and purity were assessed using a NanoDrop 2000 spectrophotometer (Thermo Fisher, USA). Reverse transcription was conducted using the PrimeScript RT Reagent Kit (RR047A, Takara, Japan) to synthesize cDNA. Quantitative real‐time PCR (RT‐qPCR) was performed using the Fast SYBR Green PCR Kit (11736059, Thermo Fisher, USA). Each reaction was run in triplicate, and GAPDH was used as the internal control. Relative gene expression levels were calculated using the 2−^ΔΔCt^ method. Each experiment was repeated three times for statistical reliability. Primer sequences were sourced from Origene and are listed in Table S1, Supporting Information.

### Statistical Analysis

Statistical analysis was conducted using GraphPad Prism 9.0. Data normality was assessed using the Shapiro–Wilk test, and homogeneity of variances was evaluated using Levene's test. Outliers were identified using Grubbs’ test and excluded where appropriate. Quantitative data are presented as mean ± standard deviation (SD). The sample size (*n*) for each experiment is specified in the figure legends.

For comparisons between two groups with normally distributed data and equal variances, unpaired two‐tailed Student's *t*‐tests were applied. For multiple‐group comparisons, one‐way ANOVA followed by Tukey's post hoc test was used. In cases of unequal variances, Welch's ANOVA and Games–Howell post hoc tests were employed. For data collected at different time points, two‐way ANOVA was applied to account for both the time and treatment factors. A *p*‐value < 0.05 was considered statistically significant unless otherwise stated. No imputation was performed. All experiments were independently repeated at least three times to ensure reproducibility.

### Ethical Statement

All animal experiments were approved by the Animal Ethics Committee of Shandong Provincial Hospital affiliated to Shandong First Medical University (No. KYLL‐2023‐405).

## Conflict of Interest

The authors declare no conflict of interest.

## Author Contributions

Q.C., X.C., and R.L. contributed equally to this work. Q.C. and X.L. designed and supervised the study. X.C. and R.L. conducted the in vitro and in vivo experiments. D.S. and J.W. were responsible for the synthesis and characterization of nanoparticles. R.F. and R.G. performed the multi‐omics analyses and data interpretation. Y.X. contributed to the histological and immunohistochemical analyses. Q.L. assisted with flow cytometry and molecular assays. X.L. provided project oversight, secured funding, and revised the manuscript. All authors participated in data analysis and manuscript preparation, and approved the final version of the manuscript.

## Supporting information



Supporting Information

## Data Availability

The data that support the findings of this study are available from the corresponding author upon reasonable request.
