# Supplementary material for: Nanoparticle‐Mediated CXCL12–CXCR4 Inhibition Reprograms Macrophages and Suppresses Gastric Carcinoma
Source: Adv Sci (Weinh). 2025 Jun 19;12(30):e00225. doi: 10.1002/advs.202500225 (PMC12376508; doi:10.1002/advs.202500225)
Supplement: Supplementary file 1 — Supporting Information [file ADVS-12-e00225-s001.docx]

**Table S1. Primer sequence.**

| **Gene** | **Item number** | **Primer sequence(5’-3’)** |
| --- | --- | --- |
| CXCL12(Mouse) | MP220217 | Forward:GGAGGATAGATGTGCTCTGGAAC |
|  |  | Reverse:AGTGAGGATGGAGACCGTGGTG |
| CXCR4(Mouse) | MP202423 | Forward:GACTGGCATAGTCGGCAATGGA |
|  |  | Reverse:CAAAGAGGAGGTCAGCCACTGA |
| CD80(Mouse) | MP201952 | Forward:CCTCAAGTTTCCATGTCCAAGGC |
|  |  | Reverse:GAGGAGAGTTGTAACGGCAAGG |
| IL-1β(Mouse) | MP206724 | Forward:TGGACCTTCCAGGATGAGGACA |
|  |  | Reverse:GTTCATCTCGGAGCCTGTAGTG |
| IL-12(Mouse) | MP206746 | Forward:ACGAGAGTTGCCTGGCTACTAG |
|  |  | Reverse:CCTCATAGATGCTACCAAGGCAC |
| IL-6(Mouse) | MP206798 | Forward:TACCACTTCACAAGTCGGAGGC |
|  |  | Reverse:CTGCAAGTGCATCATCGTTGTTC |
| CD206(Mouse) | NM_008625 | Forward:GTTCACCTGGAGTGATGGTTCTC |
|  |  | Reverse:AGGACATGCCAGGGTCACCTTT |
| IL-10(Mouse) | MP206737 | Forward:CGGGAAGACAATAACTGCACCC |
|  |  | Reverse:CGGTTAGCAGTATGTTGTCCAGC |
| GAPDH(Mouse) | MP205604 | Forward:CATCACTGCCACCCAGAAGACTG |
|  |  | Reverse:ATGCCAGTGAGCTTCCCGTTCAG |

**Table S2. KEGG enrichment pathways of 27 differential metabolites.**

| **Pathway Name** | **Match**  **Status** | ***p*** | **-log(p)** | **Holm p** | **FDR** | **Impact** |
| --- | --- | --- | --- | --- | --- | --- |
| Arginine biosynthesis | 2/14 | 0.009817 | 2.008 | 0.78539 | 0.5771 | 0.13705 |
| Pentose and glucuronate interconversions | 2/19 | 0.017854 | 1.7483 | 1 | 0.5771 | 0.24096 |
| beta-Alanine metabolism | 2/21 | 0.021641 | 1.6647 | 1 | 0.5771 | 0.10448 |
| Glutathione metabolism | 2/28 | 0.037214 | 1.4293 | 1 | 0.74429 | 0.00709 |
| Arginine and proline metabolism | 2/36 | 0.058878 | 1.23 | 1 | 0.94205 | 0.28837 |
| Tryptophan metabolism | 2/41 | 0.074188 | 1.1297 | 1 | 0.98124 | 0.01592 |
| Taurine and hypotaurine metabolism | 1/18 | 0.085859 | 1.0662 | 1 | 0.98124 | 0 |
| Histidine metabolism | 1/16 | 0.16474 | 0.78319 | 1 | 1 | 0.22131 |
| Pantothenate and CoA biosynthesis | 1/20 | 0.20174 | 0.69521 | 1 | 1 | 0.04082 |
| Alanine, aspartate and glutamate metabolism | 1/28 | 0.27115 | 0.56679 | 1 | 1 | 0 |
| Glycine, serine and threonine metabolism | 1/34 | 0.31944 | 0.49561 | 1 | 1 | 0.06902 |
| Pyrimidine metabolism | 1/39 | 0.35736 | 0.44689 | 1 | 1 | 0.01284 |
| Valine, leucine and isoleucine degradation | 1/40 | 0.36471 | 0.43806 | 1 | 1 | 0.02264 |
| Tyrosine metabolism | 1/42 | 0.37915 | 0.42118 | 1 | 1 | 0.0127 |
| Arachidonic acid metabolism | 1/43 | 0.38626 | 0.41312 | 1 | 1 | 0.01596 |
| Primary bile acid biosynthesis | 1/46 | 0.40713 | 0.39027 | 1 | 1 | 0.02285 |
| Purine metabolism | 1/71 | 0.55682 | 0.25428 | 1 | 1 | 0.00693 |
| Steroid hormone biosynthesis | 1/79 | 0.59666 | 0.22428 | 1 | 1 | 0.00678 |

**
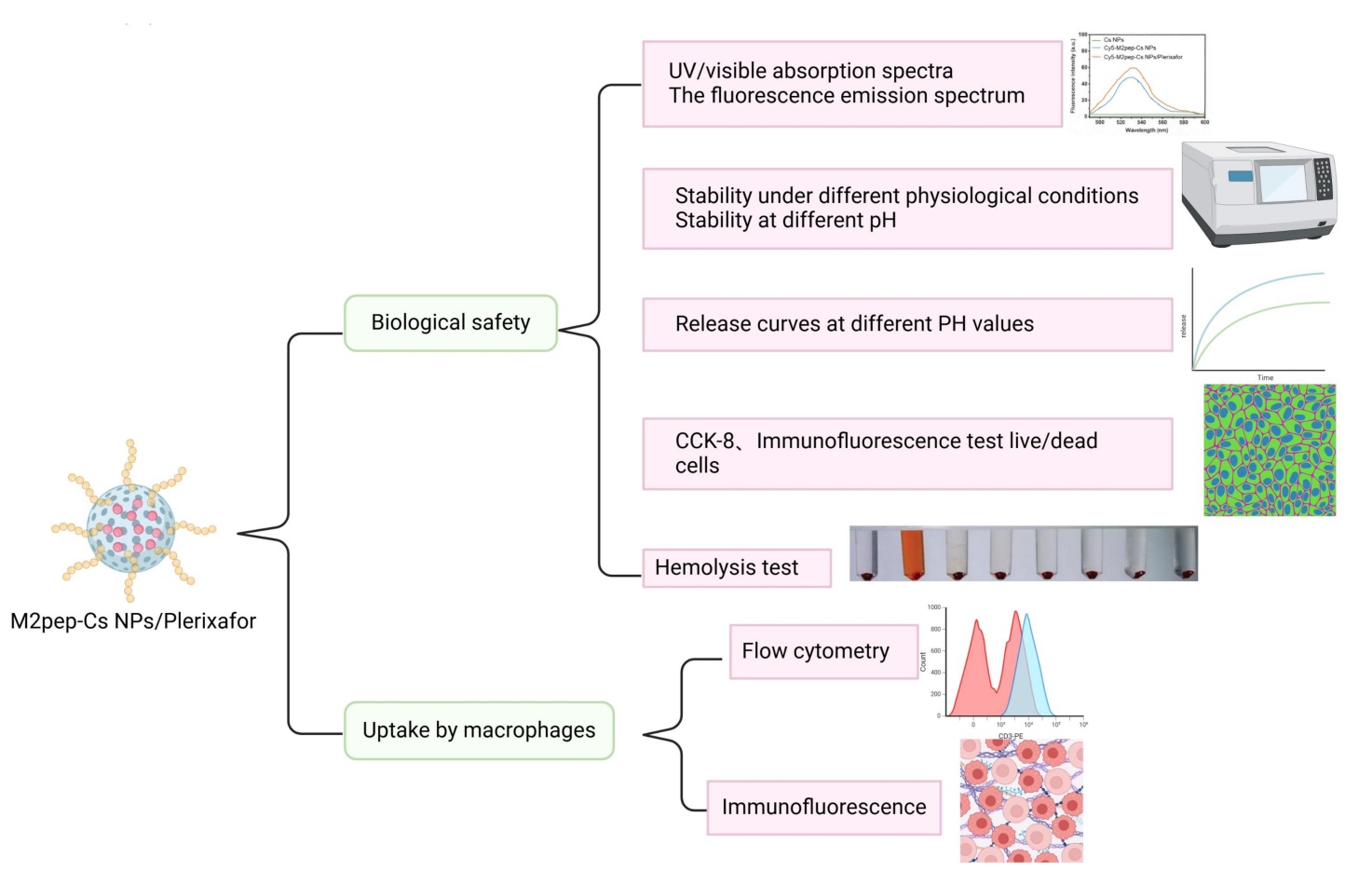
**

**Figure S1. Schematic of the biosafety evaluation and MΦ uptake of M2pep-Cs NPs/Plerixafor nanoparticles (Created by BioRender).**

**
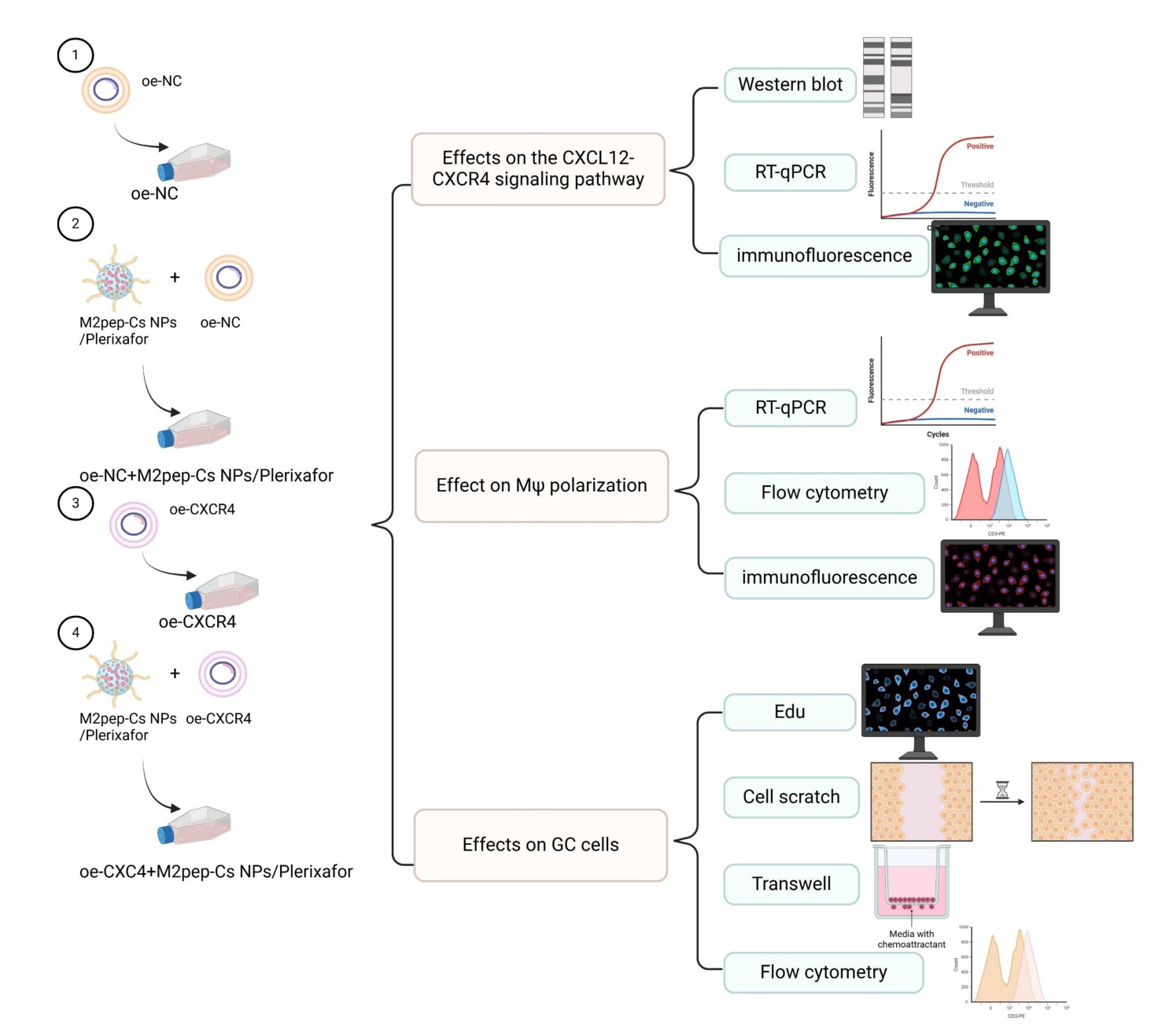
**

**Figure S2. Schematic of *in vitro* experiments illustrating the inhibitory effects of M2pep-Cs NPs/Plerixafor nanoparticles on GC (Created by BioRender).**

**
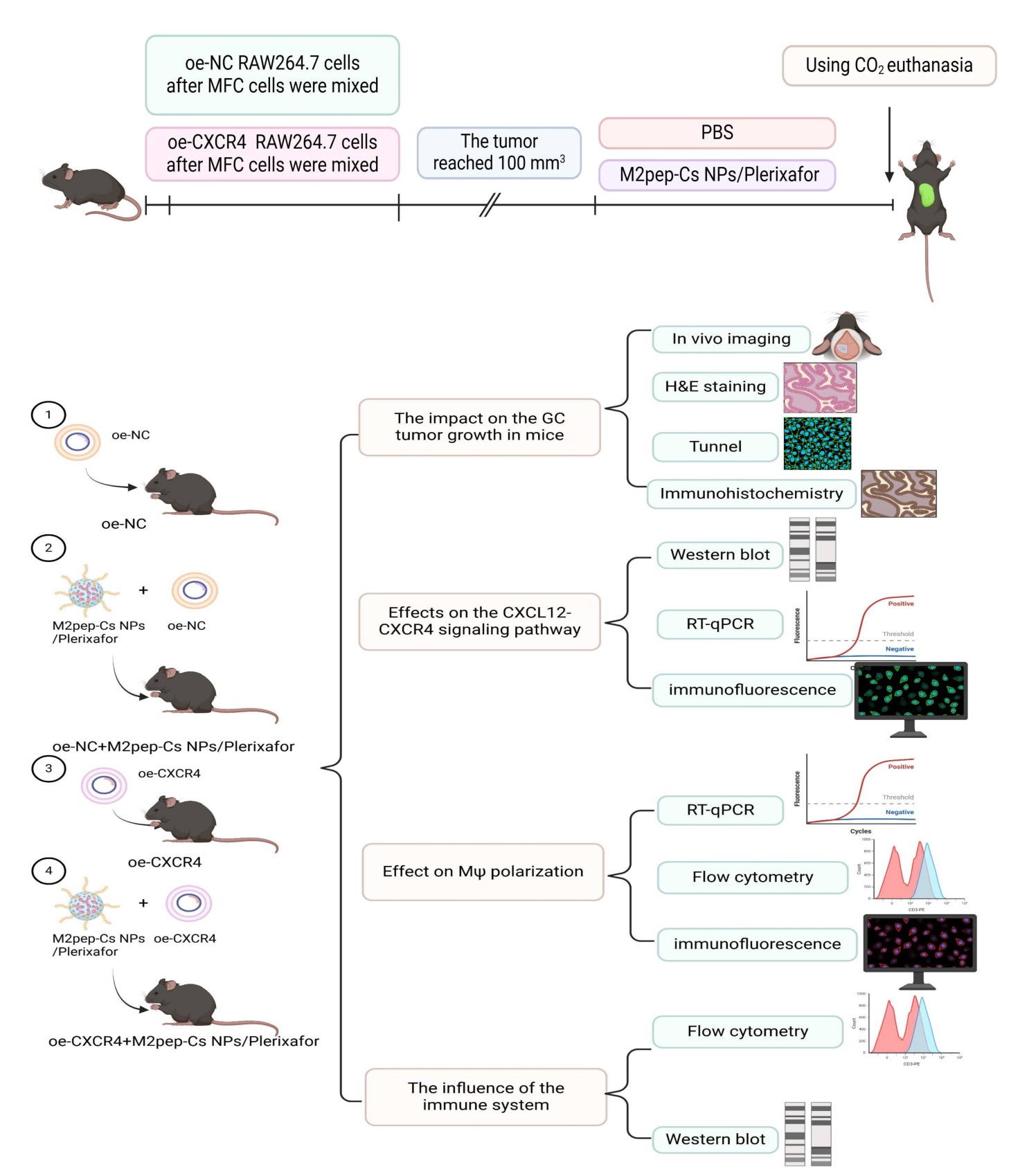
**

**Figure S3. Schematic of *in vivo* experiments demonstrating the inhibitory effects of M2pep-Cs NPs/Plerixafor nanoparticles on GC(Created by BioRender).**

**
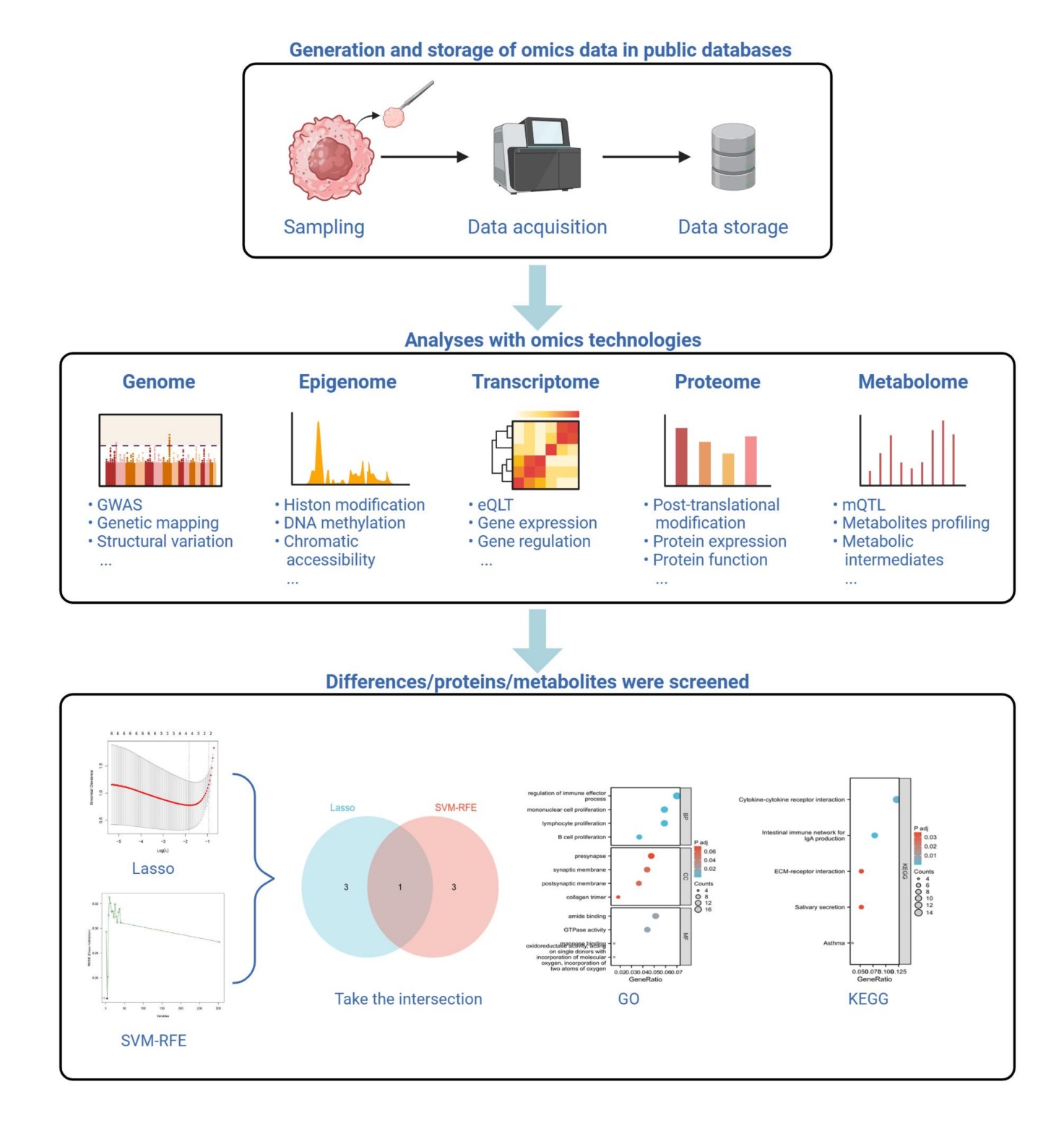
**

**Figure S4. Schematic of the multimodal analysis used to identify potential pathways through which M2pep-Cs NPs/Plerixafor nanoparticles inhibit GC(Created by BioRender).**

**
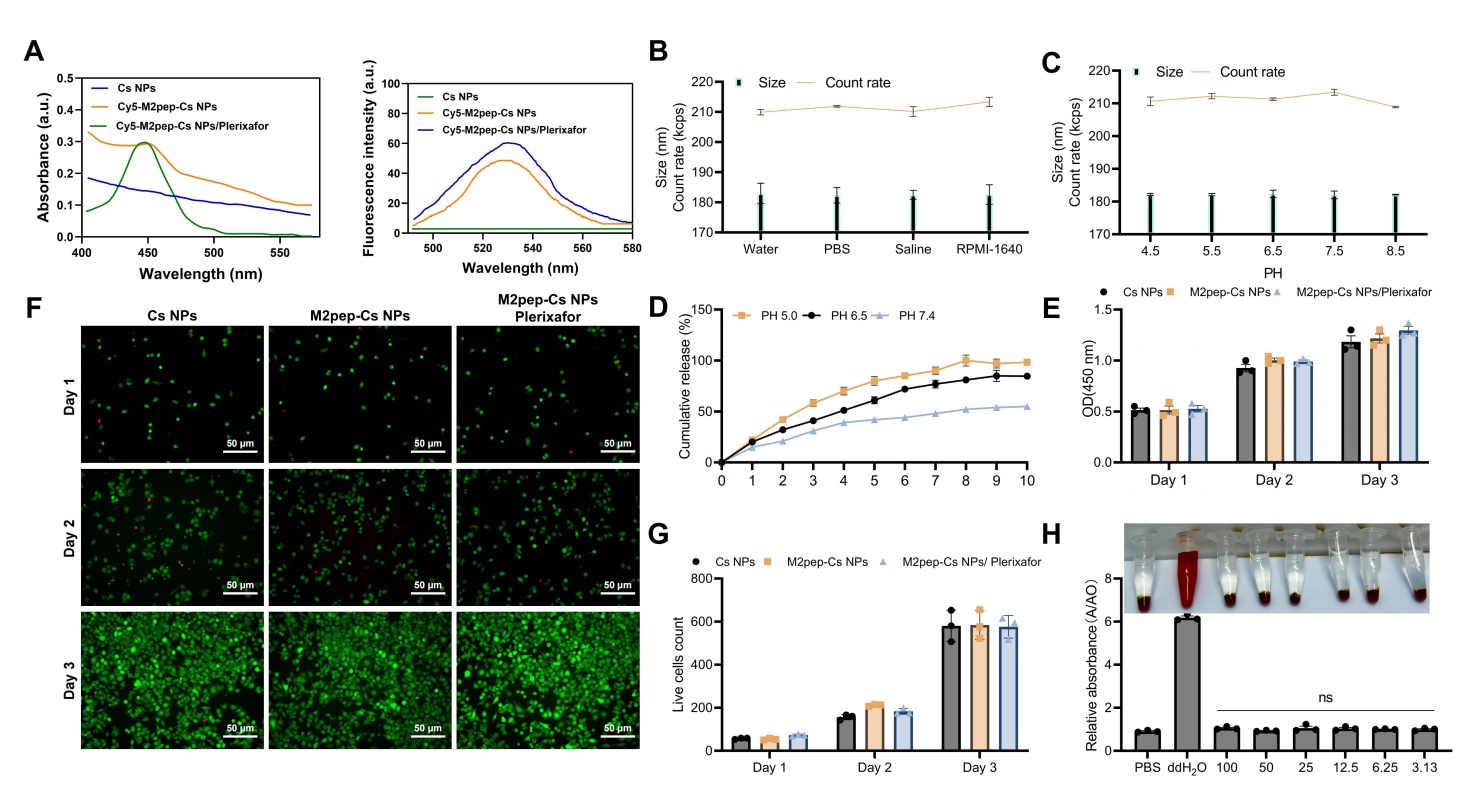
**

**Figure S5. Biosafety evaluation of M2pep-Cs NPs/Plerixafor nanoparticles.**

Note: (A) UV-visible absorption and fluorescence emission spectra of Cs NPs, M2pep-Cs NPs, and M2pep-Cs NPs/Plerixafor nanoparticles; (B) Stability of M2pep-Cs NPs/Plerixafor nanoparticles under different physiological conditions (water, PBS, saline, and RPMI 1640) assessed by DLS; (C) Stability of M2pep-Cs NPs/Plerixafor nanoparticles in buffer solutions with varying pH; (D) *In vitro* release profile of Plerixafor from M2pep-Cs NPs/Plerixafor under different pH conditions; (E) CCK-8 assay results for RAW264.7 cells at days 1, 2, and 3 in each group; (F-G) Immunofluorescence images showing LIVE/DEAD staining of RAW264.7 cells in each group, scale bar: 50 μm; (H) Hemolysis tests evaluating different concentrations of M2pep-Cs NPs/Plerixafor nanoparticles. Multiple group comparisons were conducted using one-way ANOVA, while data from different time points were analyzed using two-way ANOVA. Each experiment was repeated three times, and data are presented as mean ± standard deviation, ^ns^*p* > 0.05.

**
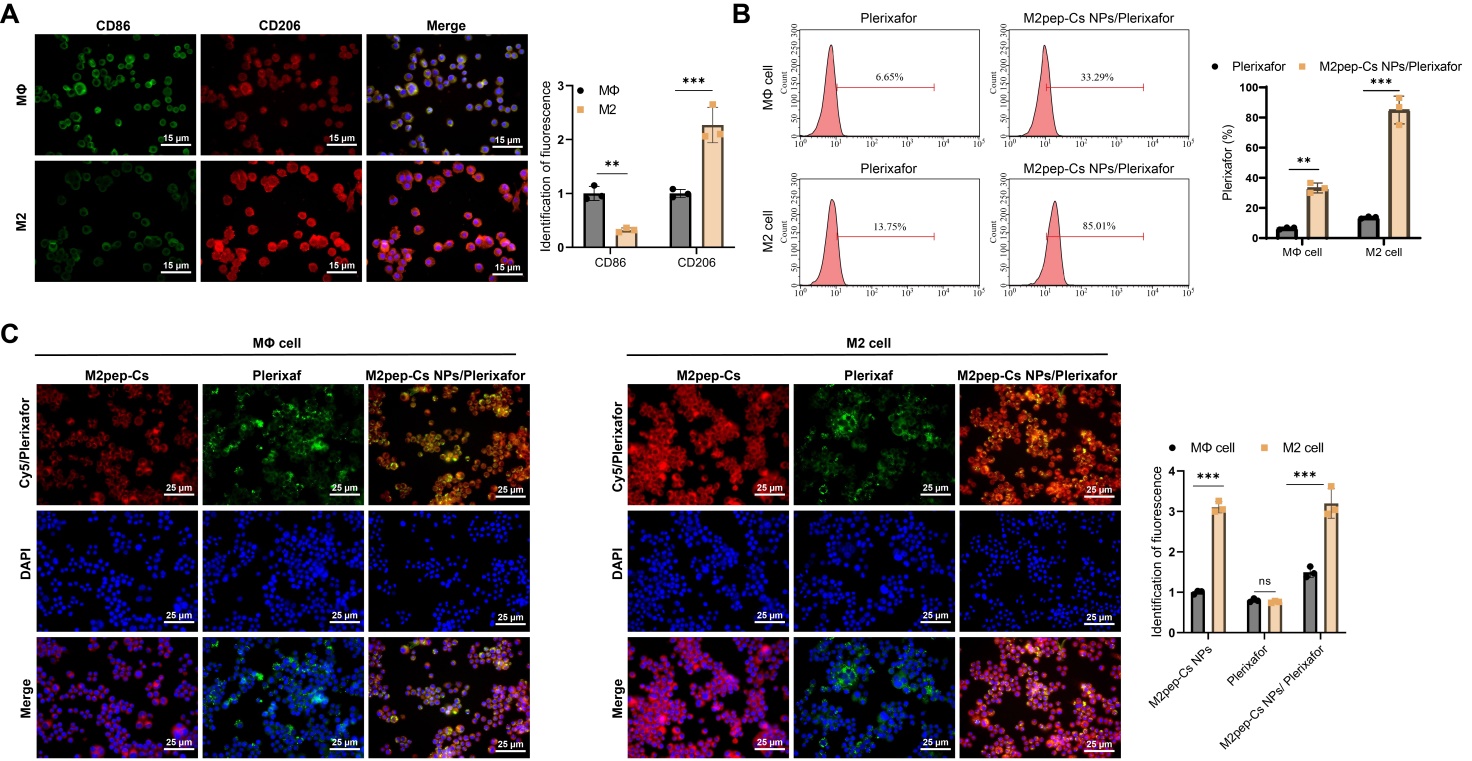
**

**Figure S6. Uptake of M2pep-Cs NPs/Plerixafor by M2 MΦ.**

Note: (A) Immunofluorescence analysis validating MΦ differentiation, scale bar: 15 μm; (B) Flow cytometry analysis of the uptake of Cs NPs, M2pep-Cs NPs, and M2pep-Cs NPs/Plerixafor nanoparticles by cells; (C) Immunofluorescence analysis of the uptake of Cs NPs, M2pep-Cs NPs, and M2pep-Cs NPs/Plerixafor nanoparticles by cells, scale bars: 25 μm. Each experiment was repeated three times, and data are presented as mean ± standard deviation. Two groups were compared using non paired t-test, ** *p* < 0.01, ****p* < 0.001.

**
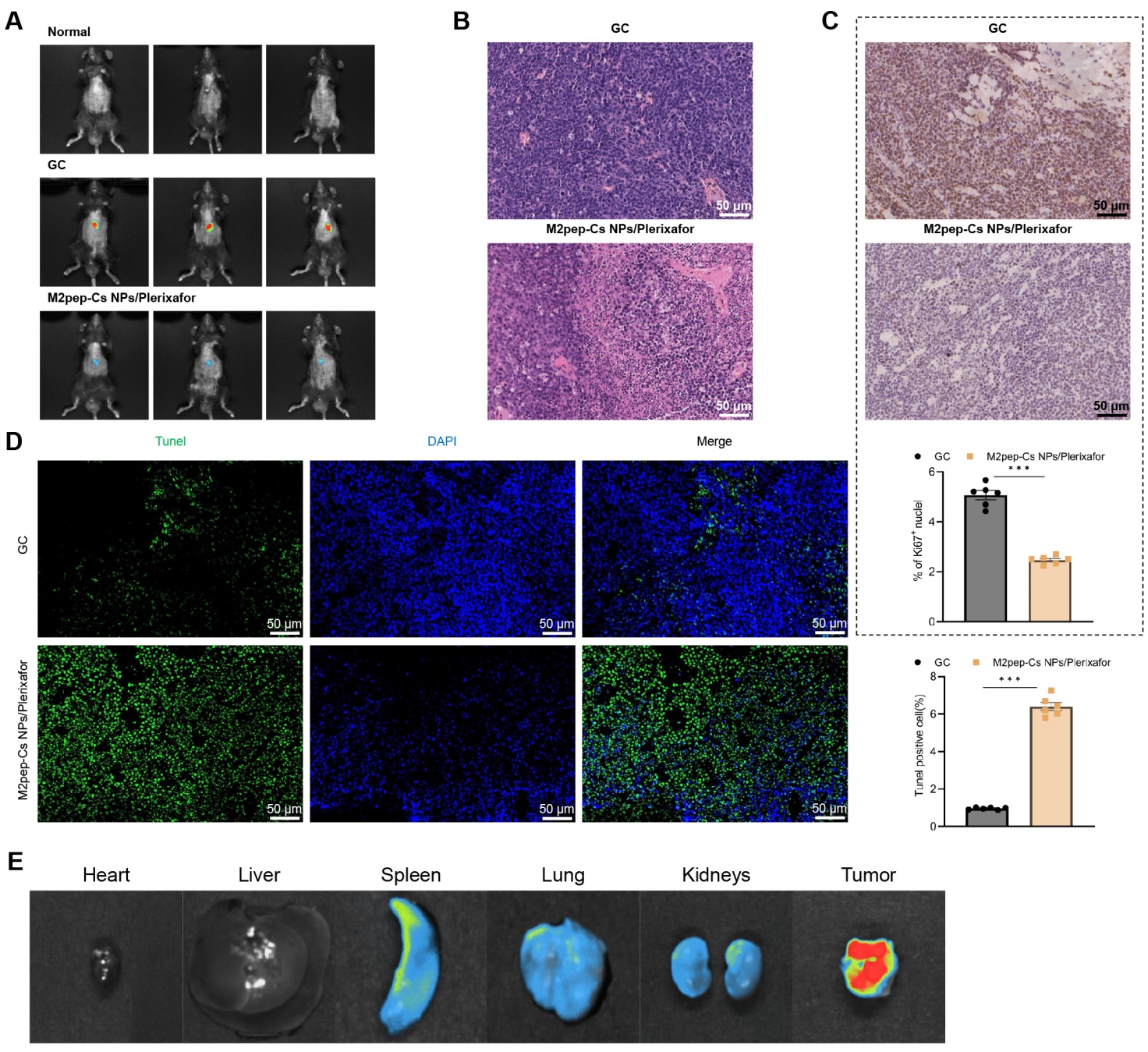
**

**Figure S7. Establishment of the GC mouse model.**

Note: (A) *In vivo* imaging showing tumor growth in different groups of mice; (B) H&E staining displaying tumor tissue morphology and structure in different groups, scale bar: 50 μm; (C) Immunohistochemistry showing Ki67 expression in tumor tissues of different groups, scale bar: 50 μm; (D) TUNEL staining showing apoptosis in tumor tissues of different groups, scale bar: 50 μm; (E) Representative fluorescence images of major organs (from left to right: heart, liver, spleen, lungs, and kidneys) and tumor tissues at seven days post-administration. Each group consisted of 6 mice, and data are presented as mean ± standard deviation. Two groups were compared using non paired t-test****p* < 0.001.

**
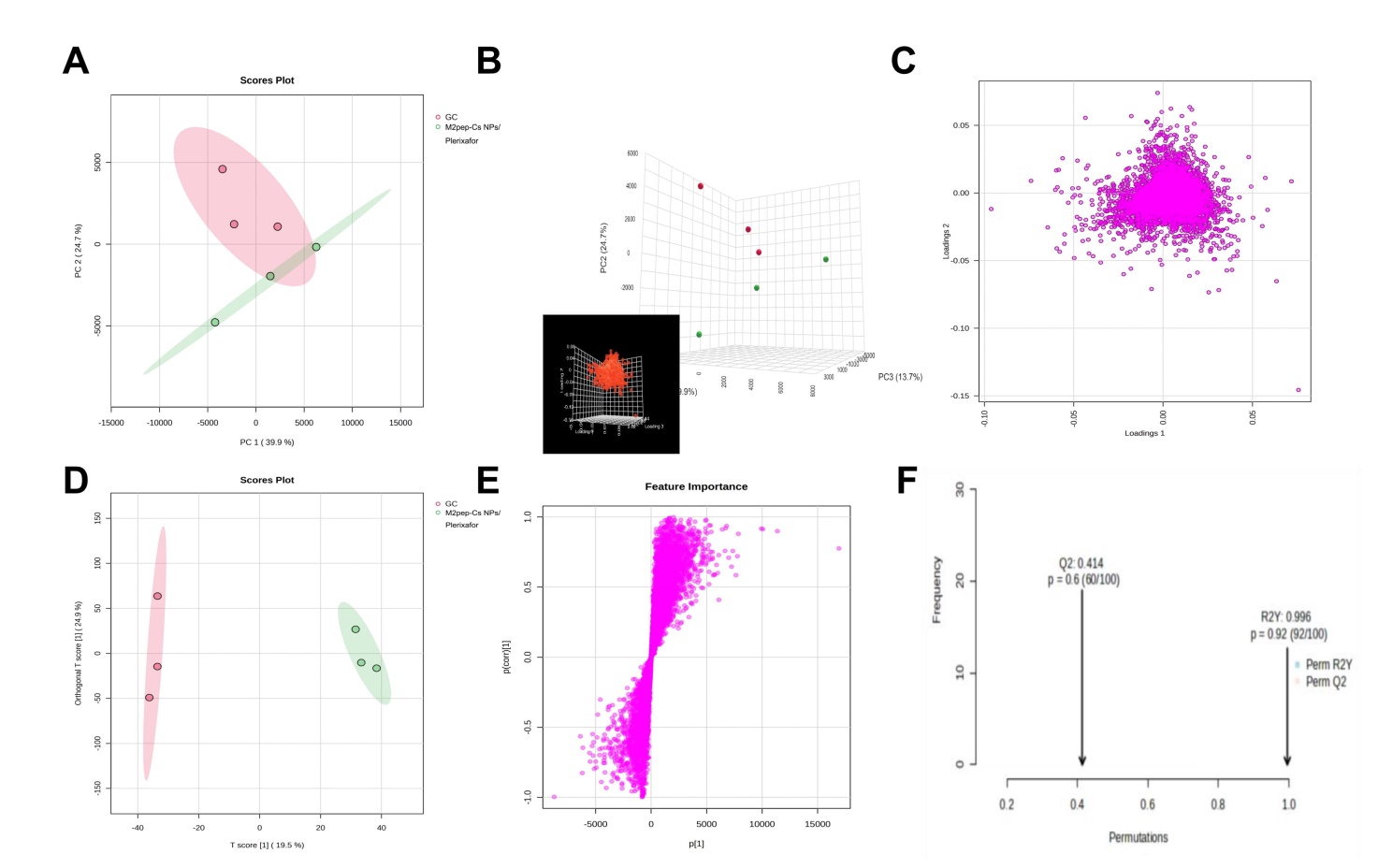
**

**Figure S8. PCA and OPLS-DA analysis based on proteomics data.**

Note: (A) PCA plot of proteomics data from the tumor tissues of 3 GC mice and 3 M2pep-Cs NPs/Plerixafor-treated mice; (B) 3D-PCA plot of proteomics data from the tumor tissues of 3 GC mice and 3 M2pep-Cs NPs/Plerixafor-treated mice; (C) Loading plot from the proteomics data of 3 GC mice and 3 M2pep-Cs NPs/Plerixafor-treated mice; (D) OPLS-DA plot of proteomics data from the tumor tissues of 3 GC mice and 3 M2pep-Cs NPs/Plerixafor-treated mice; (E) S-plot of proteomics data from the tumor tissues of 3 GC mice and 3 M2pep-Cs NPs/Plerixafor-treated mice; (F) Permutation plot of proteomics data from the tumor tissues of 3 GC mice and 3 M2pep-Cs NPs/Plerixafor-treated mice.

**
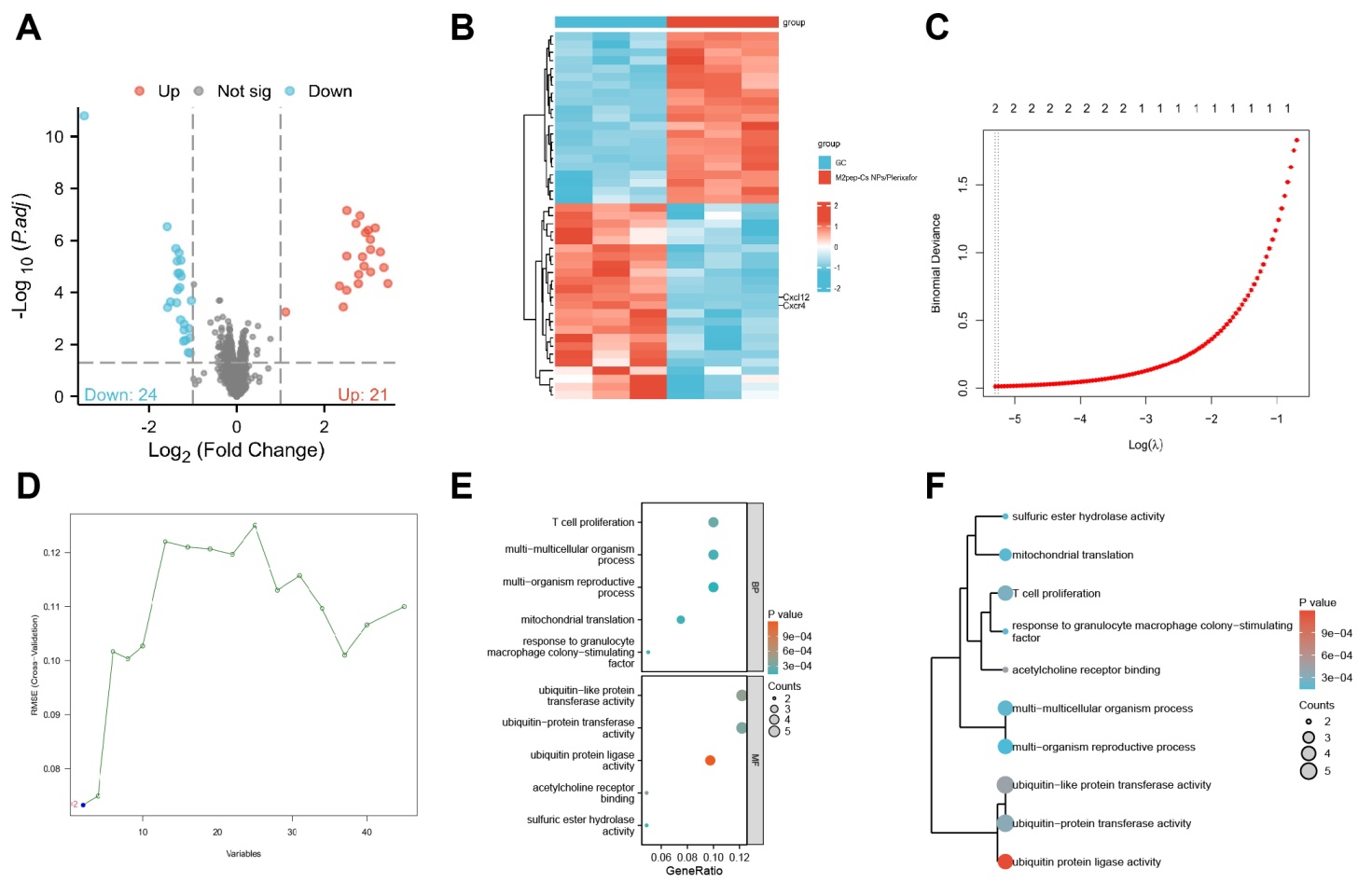
**

**Figure S9. Screening of potential pathways for GC inhibition by M2pep-Cs NPs/Plerixafor based on proteomics data.**

Note: (A) Volcano plot showing differential protein expression between tumor tissues of 3 GC mice and 3 M2pep-Cs NPs/Plerixafor-treated mice based on proteomics data; (B) Heatmap of differentially expressed proteins between tumor tissues of 3 GC mice and 3 M2pep-Cs NPs/Plerixafor-treated mice; (C) Selection of 2 key proteins using the LASSO algorithm; (D) Selection of 2 key proteins using the SVM-RFE algorithm; (E-F) GO enrichment analysis bubble chart and dendrogram of differentially expressed proteins between tumor tissues of 3 GC mice and 3 M2pep-Cs NPs/Plerixafor-treated mice.

**
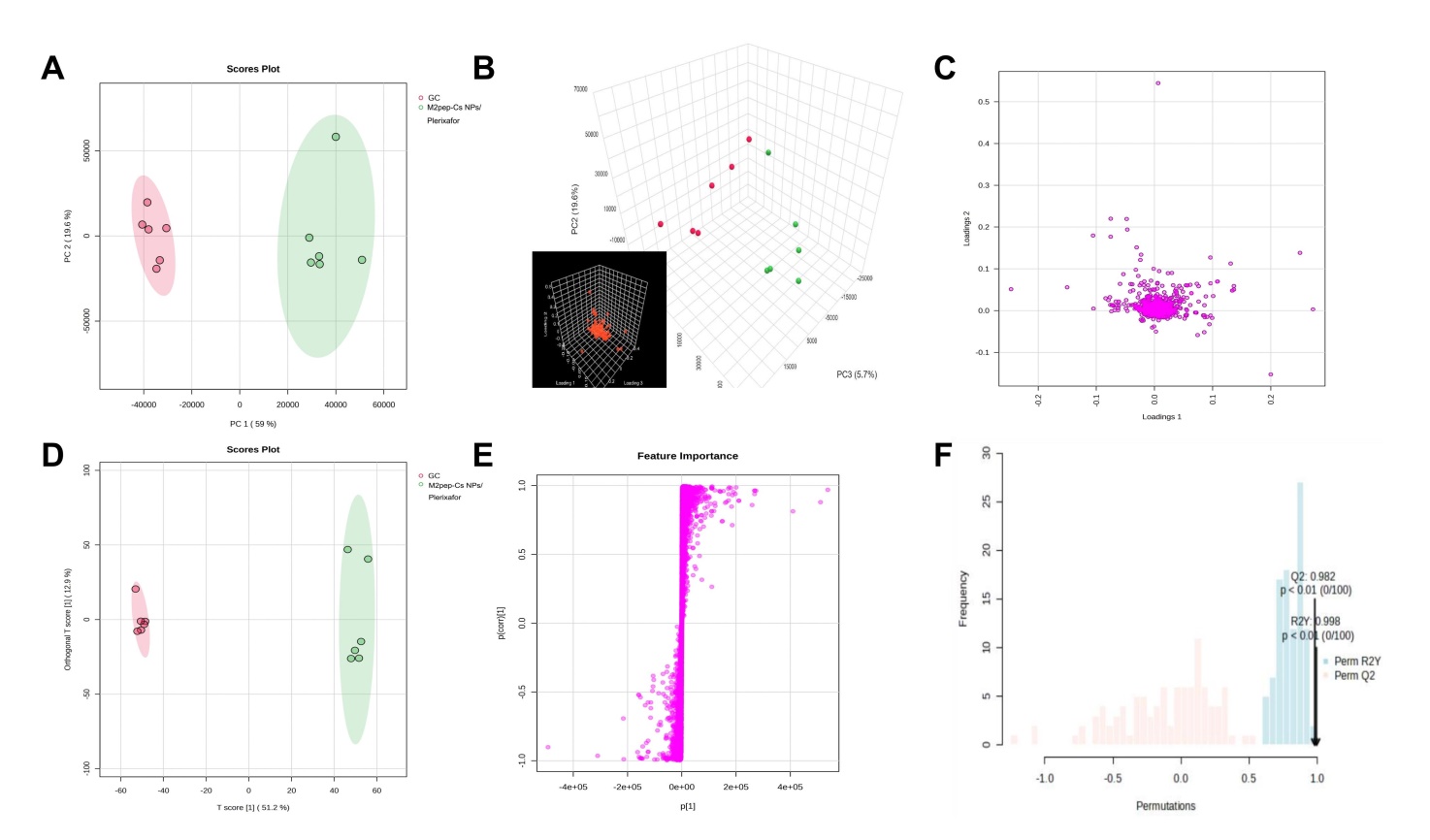
**

**Figure S10. Screening of potential pathways for GC inhibition by M2pep-Cs NPs/Plerixafor based on metabolomics data.**

Note: (A) PCA plot of metabolomics data from tumor tissues of 6 GC mice and 6 M2pep-Cs NPs/Plerixafor-treated mice; (B) 3D-PCA plot of metabolomics data from tumor tissues of 6 GC mice and 6 M2pep-Cs NPs/Plerixafor-treated mice; (C) Loading plot of metabolomics data from tumor tissues of 6 GC mice and 6 M2pep-Cs NPs/Plerixafor-treated mice; (D) OPLS-DA plot of metabolomics data from tumor tissues of 6 GC mice and 6 M2pep-Cs NPs/Plerixafor-treated mice; (E) S-plot of metabolomics data from tumor tissues of 6 GC mice and 6 M2pep-Cs NPs/Plerixafor-treated mice; (F) Permutation plot of metabolomics data from tumor tissues of 6 GC mice and 6 M2pep-Cs NPs/Plerixafor-treated mice.

**
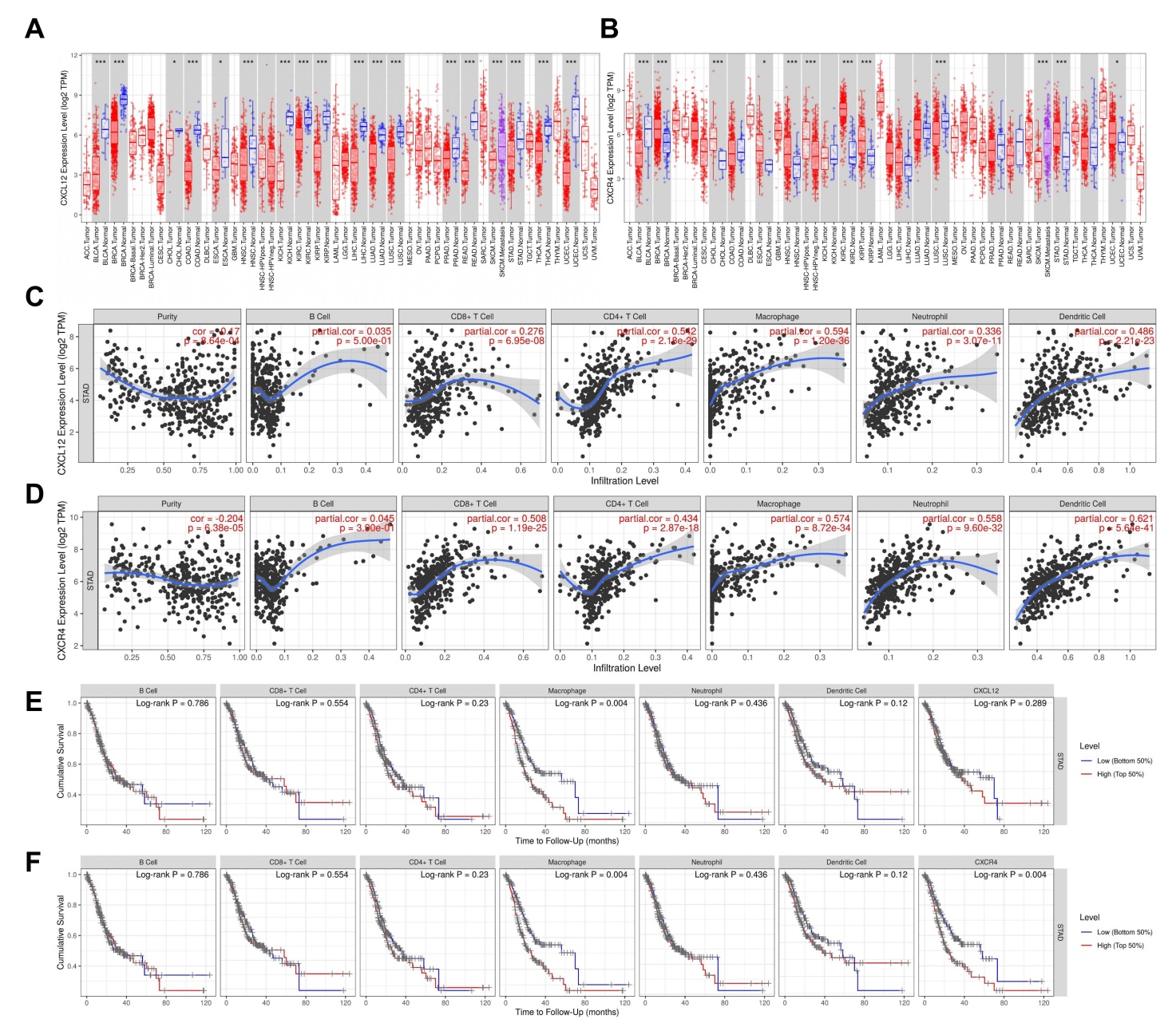
**

**Figure S11. Analysis of CXCL12 and CXCR4 in GC using the TIMER database.**

Note: (A) Expression of CXCL12 across various cancers; (B) Expression of CXCR4 across various cancers; (C) Correlation between CXCL12 and immune cell types (e.g., CD8+ T cells, MΦ); (D) Correlation between CXCR4 and immune cell types (e.g., CD8+ T cells, MΦ); (E) Survival analysis of immune cell types (e.g., CD8+ T cells, MΦ) based on high/low expression of CXCL12; (F) Survival analysis of immune cell types (e.g., CD8+ T cells, MΦ) based on high/low expression of CXCR4.

**
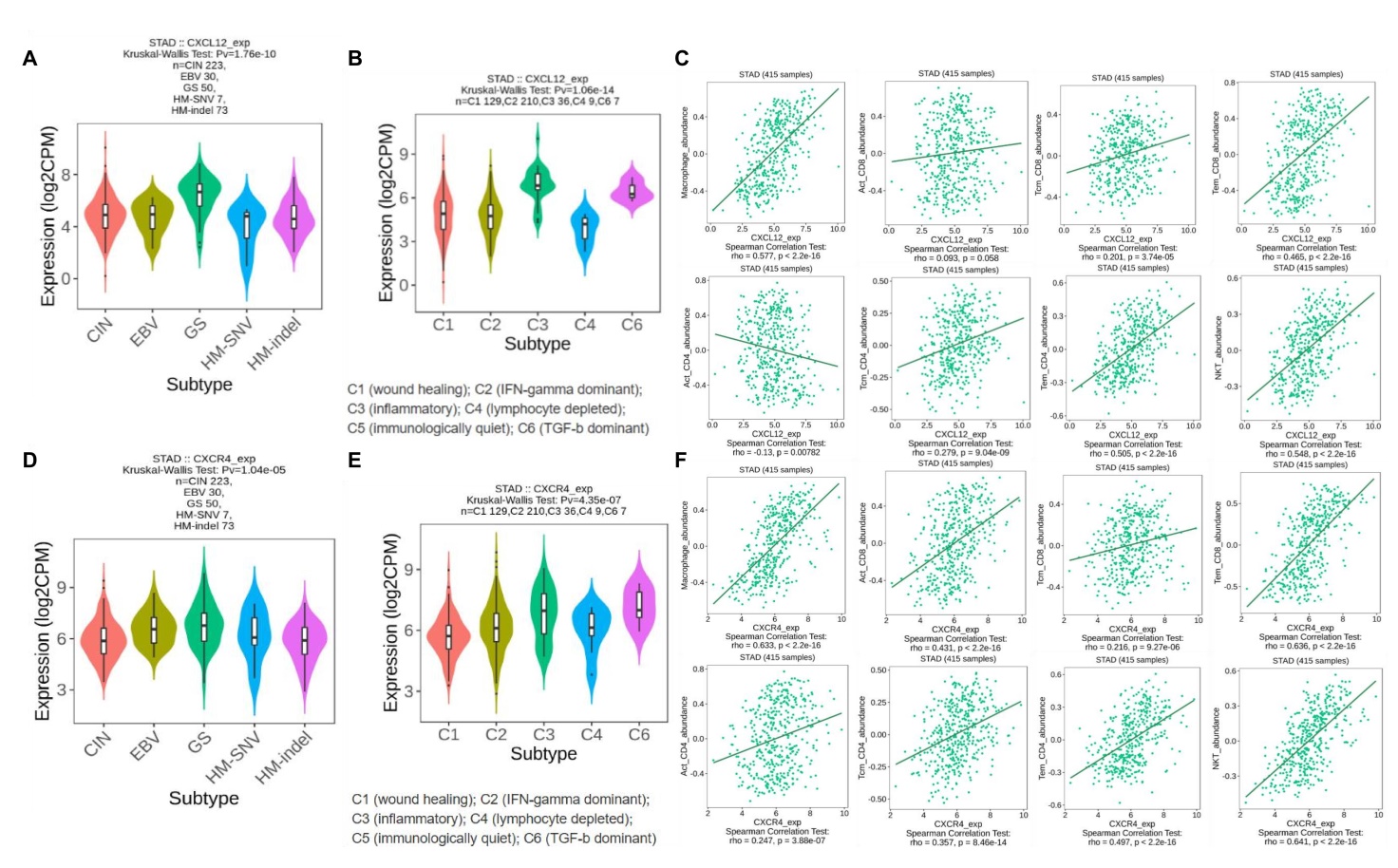
**

**Figure S12. Analysis of CXCL12 and CXCR4 in GC using the TISIDB database.**

Note: (A-B) Expression of CXCL12 in GC subtypes; (C) Correlation between CXCL12, MΦ, and different T cell types; (D-E) Expression of CXCR4 in GC subtypes; (F) Correlation between CXCR4, MΦ, and different T cell types.

**
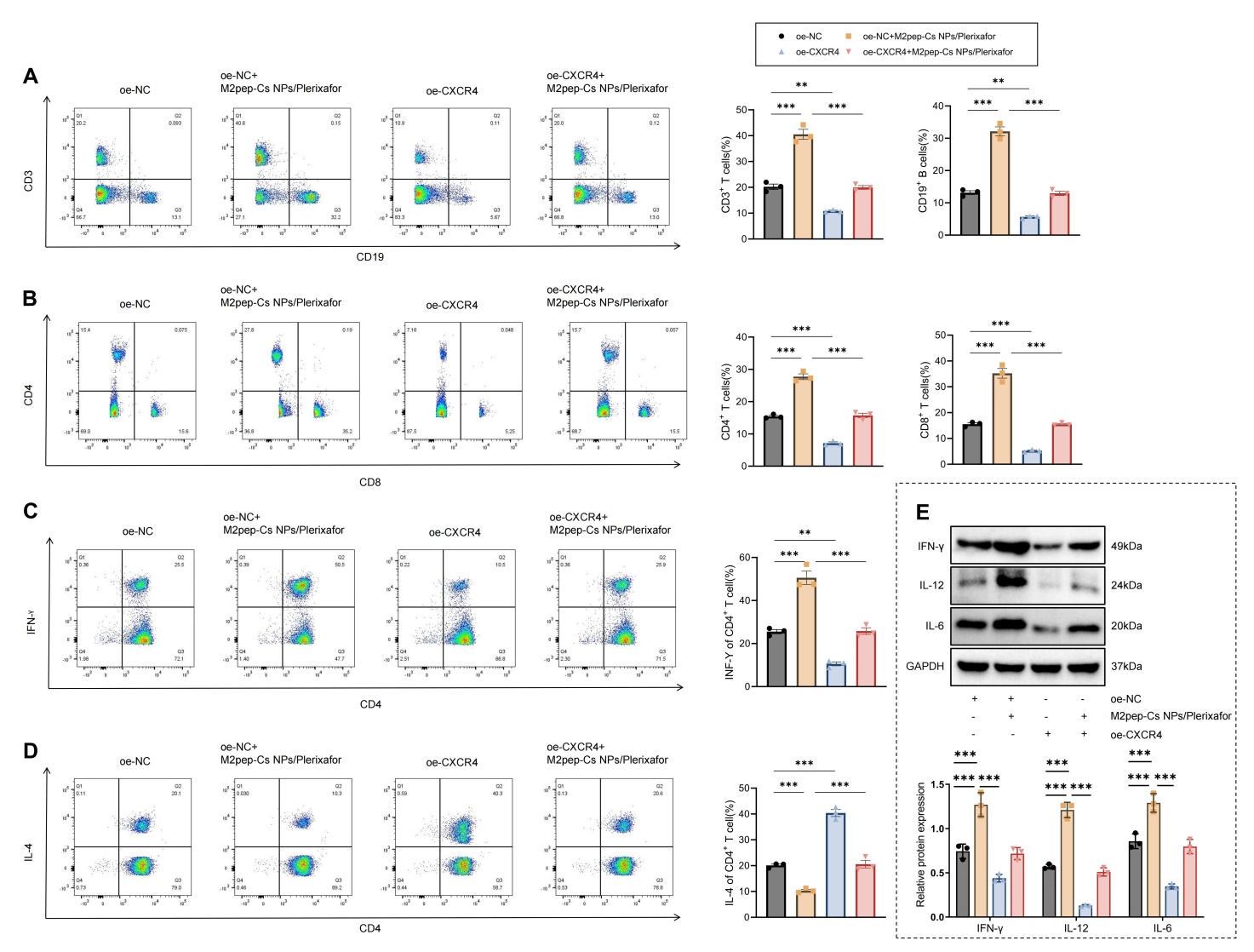
**

**Figure S13. Effects of M2pep-Cs NPs/Plerixafor nanoparticles on the immune system.**

Note: (A) Changes in T cell and B cell counts in the tumor tissues of each mouse group; (B) Changes in CD4^+^ T cell and CD8^+^ T cell counts in the tumor tissues of each mouse group; (C) Changes in Th1 cell counts in the tumor tissues of each mouse group; (D) Changes in Th2 cell counts in the tumor tissues of each mouse group; (E) Western blot analysis showing the expression levels of IFN-γ, IL-12, IL-6, and cytokines in the tumor tissues of each mouse group. Each group included 6 mice, with values presented as mean ± standard deviation. Multiple group comparisons were conducted using one-way ANOVA, *** indicates *p* < 0.001.
